# Amino acids in transmembrane helix 1 confer major functional differences between human and mouse orthologs of the polyspecific membrane transporter OCT1

**DOI:** 10.1016/j.jbc.2022.101974

**Published:** 2022-04-22

**Authors:** Marleen J. Meyer, Pascale C.F. Schreier, Mert Basaran, Stefaniia Vlasova, Tina Seitz, Jürgen Brockmöller, Barbara Zdrazil, Mladen V. Tzvetkov

**Affiliations:** 1 Department of General Pharmacology, Institute of Pharmacology, Center of Drug Absorption and Transport (C_DAT), University Medicine Greifswald, Greifswald, Germany; 2 Department of Pharmaceutical Sciences, Division of Pharmaceutical Chemistry, University of Vienna, Vienna, Austria; 3 Institute of Clinical Pharmacology, University Medical Center Göttingen, Göttingen, Germany

**Keywords:** organic cation transporter 1, SLC22A1, species differences, membrane transport, structure-function, transmembrane domain, transporter, drug transport, molecular modeling, ASP^+^, (4-(dimethylamino)styryl)-*N*-methylpyridinium, Asp474, aspartate 474, Cys36Tyr, cysteine at codon 36 to tyrosine, DPBS, Dulbecco's PBS, HBSS, Hank's buffered salt solution, HEK293, human embryonic kidney 293 cell line, IBC, isobutyryl-l-carnitine, MPP^+^, 1-methyl-4-phenylpyridinium, OCT1, organic cation transporter 1, Phe32Leu, phenylalanine at codon 32 to leucine, TEA^+^, tetraethylammonium, TMH, transmembrane helix

## Abstract

Organic cation transporter 1 (OCT1) is a membrane transporter that affects hepatic uptake of cationic and weakly basic drugs. OCT1 transports structurally highly diverse substrates. The mechanisms conferring this polyspecificity are unknown. Here, we analyzed differences in transport kinetics between human and mouse OCT1 orthologs to identify amino acids that contribute to the polyspecificity of OCT1. Following stable transfection of HEK293 cells, we observed more than twofold differences in the transport kinetics of 22 out of 28 tested substrates. We found that the β2-adrenergic drug fenoterol was transported with eightfold higher affinity but at ninefold lower capacity by human OCT1. In contrast, the anticholinergic drug trospium was transported with 11-fold higher affinity but at ninefold lower capacity by mouse Oct1. Using human–mouse chimeric constructs and site-directed mutagenesis, we identified nonconserved amino acids Cys36 and Phe32 as responsible for the species-specific differences in fenoterol and trospium uptake. Substitution of Cys36 (human) to Tyr36 (mouse) caused a reversal of the affinity and capacity of fenoterol but not trospium uptake. Substitution of Phe32 to Leu32 caused reversal of trospium but not fenoterol uptake kinetics. Comparison of the uptake of structurally similar β2-adrenergics and molecular docking analyses indicated the second phenol ring, 3.3 to 4.8 Å from the protonated amino group, as essential for the affinity for fenoterol conferred by Cys36. This is the first study to report single amino acids as determinants of OCT1 polyspecificity. Our findings suggest that structure–function data of OCT1 is not directly transferrable between substrates or species.

Organic cation transporter 1 (OCT1, official gene nomenclature name: SLC22A1) is strongly and almost exclusively expressed at the sinusoidal membrane of human hepatocytes (1, 2, 3, 4). Genetically determined loss or reduction of OCT1 activity leads to substantial changes in pharmacokinetics or hepatic concentrations of drugs in humans (2, 5, 6, 7, 8, 9). Also in mice, Oct1 knockout affects pharmacokinetics and organ concentrations of drugs and toxins (10, 11, 12).

One important characteristic of OCT1is its polyspecificity. A variety of structurally diverse compounds are OCT1 substrates (13, 14). Known substrates are clinically relevant drugs, such as sumatriptan, fenoterol, metformin, morphine, and trospium, and endogenous compounds such as thiamine (2, 5, 6, 9, 11, 14, 15, 16). The exact amino acids involved in binding and/or translocation of the various OCT1 substrates and thus conferring OCT1 polyspecificity are unclear. No crystal structure of OCT1 or a closely related transporter is available, and the homology models that exist are based on proteins with less than 20% amino acid identity to OCT1 (17). Although these models have been successfully employed to identify substances interacting with OCT1 (18, 19), data obtained by site-directed mutagenesis of single amino acids are essential to generate hypotheses about the mechanism of these interactions.

The so far available structure-to-function data about OCT1 are based on mutagenesis experiments using rat Oct1 and model substrates tetraethylammonium (TEA^+^) and 1-methyl-4-phenylpyridinium (MPP^+^) (20, 21, 22, 23, 24, 25). Applying these models, aspartate 474 (Asp474 in humans corresponds to Asp475 in rodents) has been identified as essential for pairing with the positive charge of TEA^+^, MPP^+^, and other model OCT1 substrates (20). This knowledge has been confirmed in homology models (26), and postulating this interaction for other substrates was successfully used to identify novel OCT1 inhibitors (18). However, OCT1 is thought to have multiple binding sites that may overlap between substrates (27, 28). Amino acids beyond Asp474 were experimentally identified to be involved in substrate interaction but using only model substrates in rat Oct1 (23, 25, 27, 28).

While differences in the organ expression of OCT1 between human and mouse are well documented (29, 30, 31, 32), much less is known about differences in transport kinetics and substrate selectivity. Differences in substrate selectivity between human and mouse have been reported before (33). The protein sequence of mouse and human OCT1 differs in 124 amino acids, and it is unclear which of these confer differences in OCT1 transport. Dresser *et al.* (33) performed only proof-of-principle experiments using a limited number of substrates without addressing in details the underlying mechanisms of the species differences.

The aim of this study was to assess differences in OCT1 function between the species by comparing the transport kinetics of a large number of substrates between human and mouse OCT1. Moreover, we aimed to identify regions or even single amino acids in the OCT1 protein that confer the species differences. The results from this study should help to identify new amino acids involved in substrate interaction of OCT1 and to better understand the mechanisms conferring OCT1 polyspecificity.

## Results

**Figure 1:**
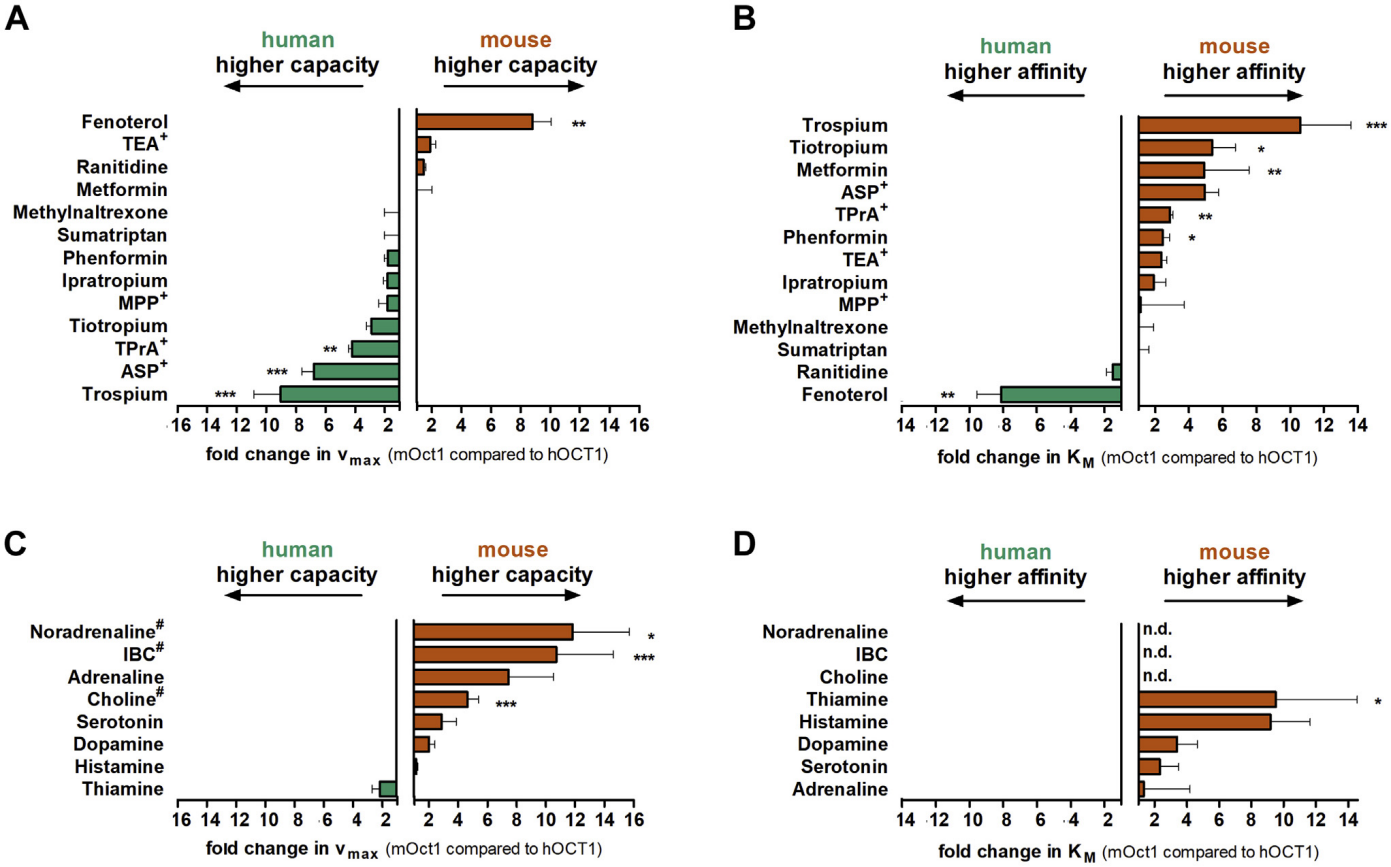
**Comparison of transport capacity (*v***_**max**_**) and affinity (*K***_***M***_**) between human and mouse OCT1 orthologs.** Shown are the fold change in *v*_max_ (*A* and *C*) and *K*_*M*_ (*B* and *D*) between human and mouse OCT1 for 13 drugs and model compounds (*A* and *B*) and eight endogenous compounds (*C* and *D*). For isobutyryl-l-carnitine (IBC), choline, and noradrenaline, the fold change at the second (IBC) or at the highest concentration, respectively, is shown because the data for human OCT1 could not be fit to the Michaelis–Menten equation (*C*). *K*_*M*_ could not be determined (ND). Shown are means and standard deviation of at least three independent experiments. ∗*p* < 0.05, ∗∗*p* < 0.01, and ∗∗∗*p* < 0.001 in a Student’s *t* test with Bonferroni correction. Data of IBC, metformin, and thiamine have previously been published (34, 35). OCT1, organic cation transporter 1.

Initially, we compared the uptake of nine drugs, four model compounds, and eight endogenous substances between human and mouse OCT1 using human embryonic kidney 293 (HEK293) cells stably transfected to overexpress mouse or human orthologs. The stably transfected cells used here were shown before to have comparable expression levels and localization of human and mouse OCT1 (34). Concentration-dependent measurements showed strong differences in the uptake kinetics between human and mouse OCT1 orthologs (Fig. 1 and Table 1). The differences were observed both in the capacity (*v*_max_) and in the affinity (*K*_*M*_).

**Table 1:** Kinetic parameter of OCT1-mediated substrate uptake by human and mouse OCT1

Substrate	*v*_max_ ± SD (pmol × min^−1^ × mg protein^−1^)		*K*_*M*_ ± SD (μM)		CL_int_ ± SD (μl × min^−1^ × mg protein^−1^)	
	hOCT1	mOct1	hOCT1	mOct1	hOCT1	mOct1
Drugs						
*Fenoterol*	90.5 ± 19.3	780.8 ± 68.5	0.87 ± 0.19	6.90 ± 0.56	104 ± 2.17	113 ± 5.08
Formoterol	434 ± 111	1458 ± 317	13.2 ± 3.71	9.81 ± 2.26	33.0 ± 2.93	150 ± 21.4
Ipratropium	349 ± 53.9	192.9 ± 13.1	10.7 ± 0.70	6.06 ± 1.76	32.5 ± 3.06	33.8 ± 10.3
Metformin	14,703 ± 4346	17,496 ± 7097	2198 ± 1154	491 ± 155	7.85 ± 3.90	37.0 ± 16.1
Methylnaltrexone	495 ± 142	414 ± 130	10.0 ± 2.76	8.61 ± 3.12	51.1 ± 13.5	51.6 ± 18.6
Orciprenaline	1598 ± 34.4	4235 ± 773	530 ± 44.9	89.8 ± 9.34	3.03 ± 0.22	47.6 ± 11.1
Phenformin	1957 ± 418	1072 ± 182	18.7 ± 2.64	7.53 ± 0.63	106 ± 22.8	143 ± 26.2
Pirbuterol	2017 ± 567	1849 ± 308	29.2 ± 12.2	26.5 ± 12.5	73.5 ± 24.2	76.3 ± 21.0
Ractopamine	336 ± 24.5	481 ± 77.1	2.17 ± 0.59	2.54 ± 0.83	162 ± 41.0	207 ± 90.5
Ranitidine	4095 ± 1160	5797 ± 947	55.7 ± 4.63	84.4 ± 27.0	75.0 ± 27.8	74.1 ± 27.8
Ritodrine	153 ± 22.1	737 ± 90.1	0.68 ± 0.12	6.34 ± 0.89	226 ± 8.36	116 ± 3.32
Salbutamol	1382 ± 43.6	1975 ± 117	395 ± 56.8	52.2 ± 7.27	3.55 ± 0.52	38.2 ± 4.28
Sumatriptan	4179 ± 1571	3252 ± 1263	65.9 ± 15.1	65.7 ± 17.0	63.6 ± 18.1	50.5 ± 16.8
Terbutaline	800 ± 244	2952 ± 1078	197 ± 54.1	60.4 ± 18.7	3.81 ± 1.28	48.7 ± 7.82
Tiotropium	836 ± 322	285 ± 87.1	7.82 ± 1.77	1.47 ± 0.12	106 ± 28.1	193 ± 55.7
*Trospium*	1411 ± 287	163 ± 45.1	17.0 ± 5.46	1.81 ± 1.03	86.7 ± 18.3	102 ± 29.3
Model substrates						
ASP^+^^a^	361,671 ± 2974	53,802 ± 7155	63.0 ± 15.3	12.8 ± 2.97	5981 ± 1583	4260 ± 436
MPP^+^	3112 ± 419	1910 ± 450	71.1 ± 17.3	24.4 ± 7.95	44.9 ± 8.69	80.8 ± 12.1
TEA^+^	4207 ± 1511	7764 ± 2883	749 ± 438	305 ± 141	6.31 ± 2.45	27.6 ± 9.72
TPrA^+^	1822 ± 97.9	433 ± 38.1	11.4 ± 0.58	3.97 ± 0.42	160 ± 9.50	110 ± 14.2
Endogenous compounds						
Adrenaline	1123 ± 558	7547 ± 2250	558 ± 411	242 ± 38.6	2.69 ± 1.34	31.2 ± 8.16
Choline	ND	16,564 ± 5901	ND	1286 ± 308	ND	13.0 ± 4.54
Dopamine	718 ± 305	1714 ± 322	674 ± 194	285 ± 3.25	1.04 ± 0.15	6.00 ± 1.13
Histamine	5124 ± 2406	5961 ± 2977	4675 ± 2319	497 ± 136	1.27 ± 0.83	11.9 ± 5.08
IBC	ND	8366 ± 1705	ND	1377 ± 356	ND	6.13 ± 0.33
Noradrenaline	ND	6068 ± 3279	ND	409 ± 60.7	ND	11.6 ± 5.17
Serotonin	6183 ± 2193	16,162 ± 2756	663 ± 334	257 ± 53.3	10.0 ± 2.83	63.9 ± 9.10
Thiamine	8261 ± 3720	3712 ± 1031	1057 ± 341	143 ± 96.3	7.71 ± 1.75	34.1 ± 18.3

Abbreviations: CL_int_, intrinsic clearance calculated as *v*_max_/*K*_*M*_ for each single experiment; hOCT1, human OCT1; mOct1, mouse Oct1; ND, not determined (data could not be fit to Michaelis–Menten equation; RFU, relative fluorescent unit.

a *v*_max_ in RFU × min^−1^ × mg protein^−1^.

**Figure 2:**
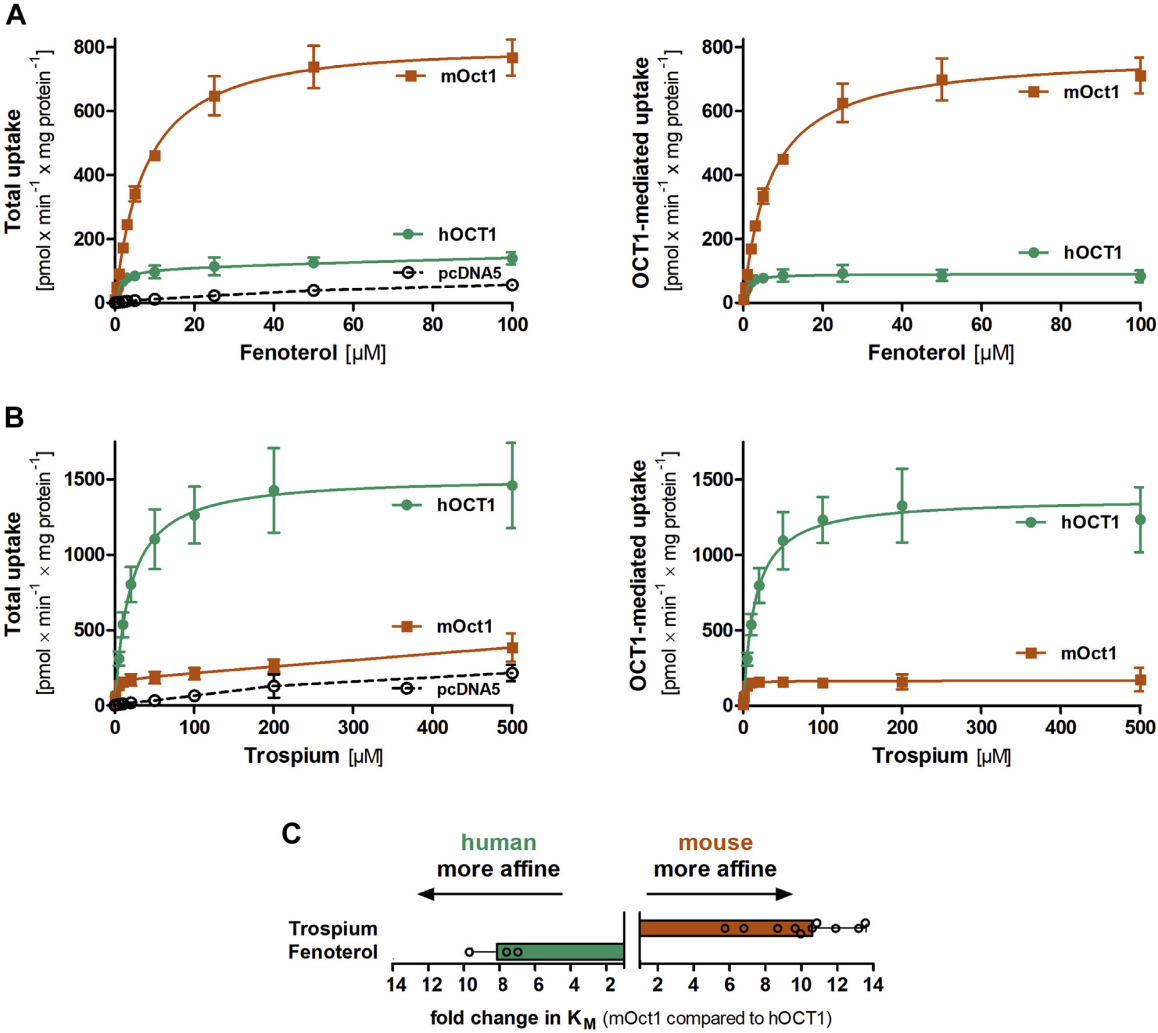
**Comparative characterization of fenoterol and trospium uptake between human and mouse OCT1 orthologs.** HEK293 cells stably transfected to overexpress human (*green*) and mouse (*red*) OCT1 were incubated for 2 min with increasing concentrations of fenoterol (*A*) or trospium (*B*). The total uptake was shown for the overexpressing and control cells (*left-hand side* of *A* and *B*). OCT1-mediated uptake (*right-hand side* of *A* and *B*) was calculated by subtracting the uptake of control cells (pcDNA5) from the uptake of OCT1-overexpressing cells. *C*, shows the fold change in *K*_*M*_ based on the data in *A* and *B*. Shown are means and standard deviation of at least three independent experiments. HEK293, human embryonic kidney 293 cell line; OCT1, organic cation transporter 1.

Based on the observed differences in affinity (Fig. 1*B*), the substrates tested could be stratified into three groups. The first group comprises substrates with a significantly higher affinity of human than of mouse OCT1. This group is represented by fenoterol. Fenoterol was transported with an 8.1-fold higher affinity by human than by mouse OCT1 (Figs. 2, *A* and *C*, S1 and Table 1). This was accompanied with an 8.8-fold lower capacity, making human OCT1 a high-affinity low-capacity transporter of fenoterol and mouse Oct1 vice versa (Fig. 2, *A* and *C*). The second group comprises substrates with a higher affinity of mouse than of human OCT1. This group not only is represented by trospium but also includes tiotropium, metformin, 4-(4-(dimethylamino)styryl)-*N*-methylpyridinium (ASP^+^), and tetrapropylammonium (Fig. 1). Human OCT1 showed a 9.0-fold higher capacity and 10.6-fold lower affinity for trospium than mouse Oct1 (Fig. 1, *A* and *B*, Table 1 and Fig. 2, *B* and *C*). Thus, human OCT1 is a low-affinity high-capacity transporter, and mouse Oct1 is a high-affinity low-capacity transporter of trospium (Fig. 2). Finally, we observed a third group comprising substrates with no substantial differences in affinity, such as phenformin, TEA^+^, ipratropium, MPP^+^, methylnaltrexone, sumatriptan, and ranitidine (Fig. 1).

For all endogenous compounds tested, mouse Oct1 had higher affinity compared with human OCT1. Furthermore, noradrenaline, isobutyryl-l-carnitine (IBC), and choline lacked any saturable transport by human OCT1 (Table 1 and Fig. S2). Interestingly, the higher affinity of mouse Oct1 was not related with lower capacity. In contrast, except for thiamine, mouse Oct1 had both higher affinity and higher capacity for the endogenous substrates tested.

### Using chimeric constructs to identify regions conferring differences in substrate selectivity between human and mouse OCT1

**Figure 3:**
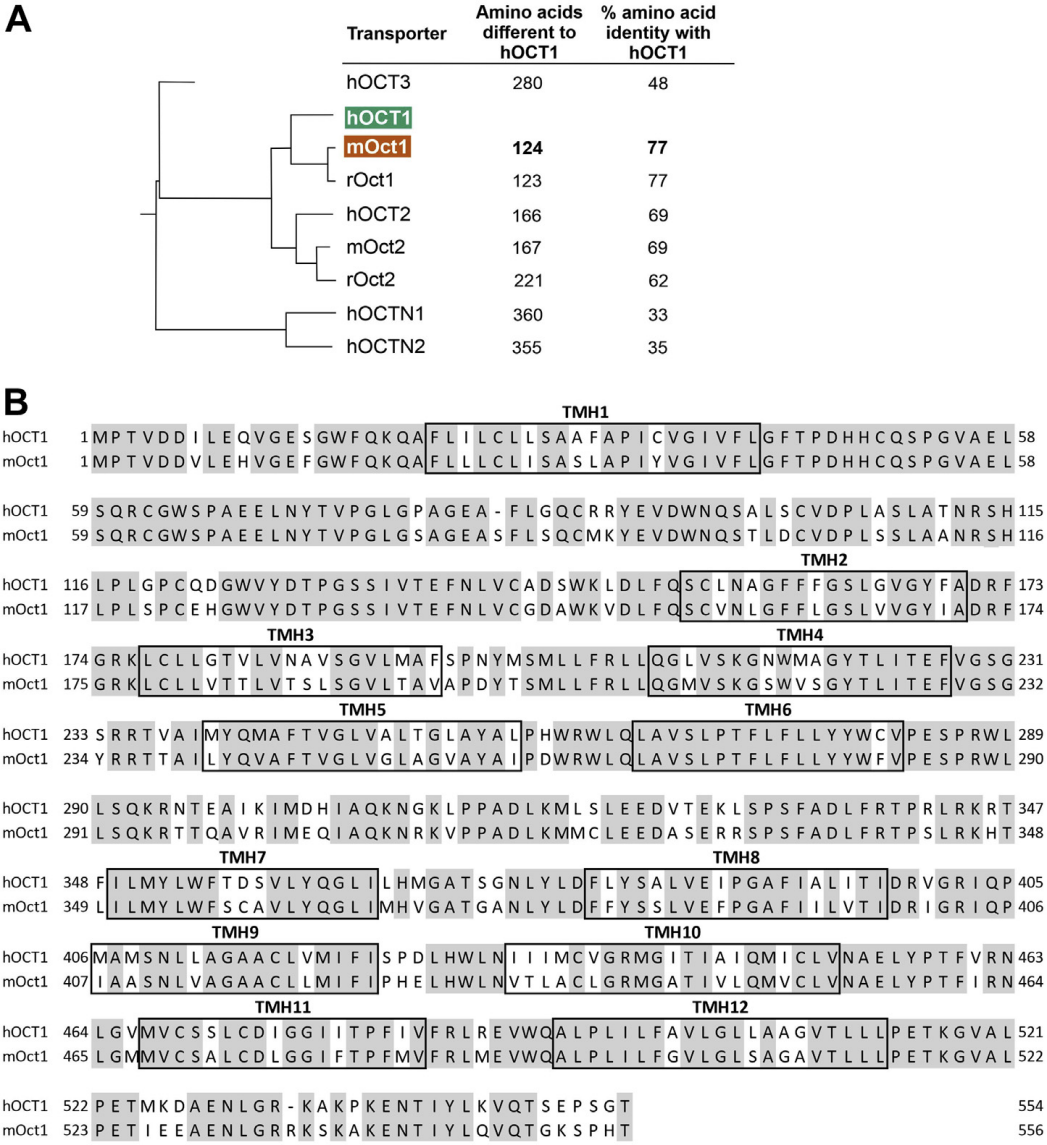
**Sequence identity of human and mouse OCT1.***A*, shows the phylogenetic tree of human OCT1 and its human and rodent orthologs and paralogs. Given are also the number of different amino acids and percentage sequence identity. Protein alignment of human and mouse OCT1 using clone manager suite, version 6.0 and BLOSUM62 algorithm (*B*). Identical amino acids are shaded in *gray*. Transmembrane helices (TMHs) 1 to 12 are shown in *boxes*. OCT1, organic cation transporter 1.

**Figure 4:**
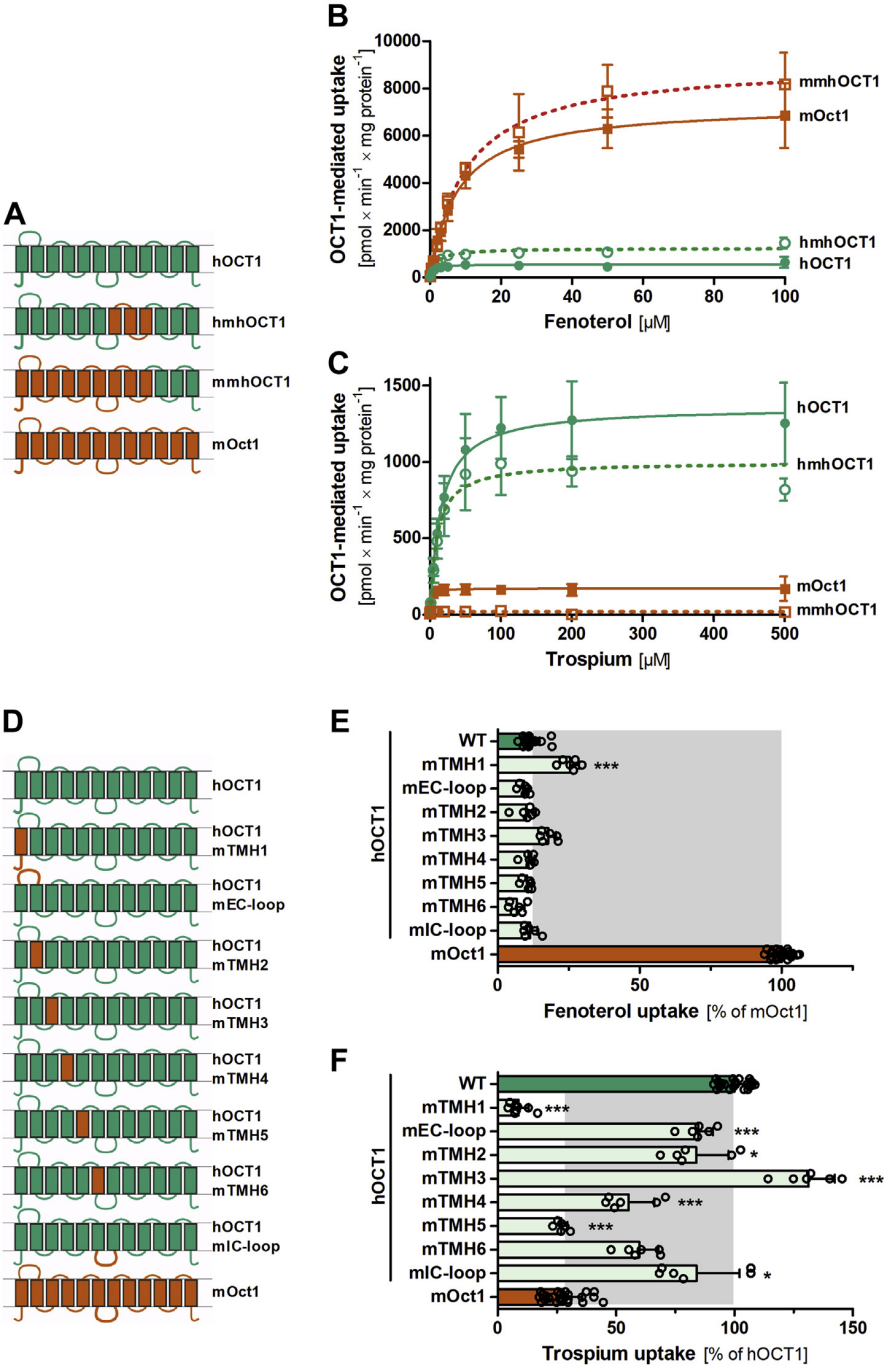
**Using human–mouse chimeric OCT1 constructs for identifying regions conferring differences in fenoterol and trospium uptake between human and mouse OCT1.***A* and *D*, schematic representation of human–mouse chimeric OCT1 constructs with colors indicating the transmembrane helices (TMHs) or loops of either human (*green*) or mouse origin (*red*). *A*–*C*, schematic representation of the low-resolution chimeric OCT1 constructs (*A*) and their effect on fenoterol (*B*) and trospium uptake (*C*). *D* and *E*, schematic representation of high-resolution chimeric OCT1 constructs with human OCT1 carrying single TMH substitutions of each of the first six TMHs, the large extracellular (EC), or the large intracellular (IC) loop of mouse Oct1 (*D*) and their effect on fenoterol (*E*) and trospium (*F*) uptake. HEK293 cells stably (*B* and *C*) or transiently (*E* and *F*) overexpressing OCT1 were incubated with increasing concentrations (*B* and *C*) or 50 μM fenoterol (*E*) or 200 μM trospium (*F*) for 2 min. OCT1-mediated uptake was calculated by subtracting the uptake of control cells (pcDNA5) from the uptake of OCT1-overexpressing cells (*E* and *F*) and related to the uptake by human (*green*) and mouse (*red*) wildtype OCT1. Shown are means and standard deviation of at least three independent experiments. ∗∗∗*p* < 0.001, ∗*p* < 0.05 compared with human OCT1 in a Tukey’s post hoc analysis following one-way ANOVA. HEK293, human embryonic kidney 293 cell line; OCT1, organic cation transporter 1.

Next, we looked for the mechanisms underlying the major differences in the uptake of human and mouse orthologs. Human and mouse OCT1 differ in 124 amino acids (23% of their sequence). The different amino acids are evenly distributed throughout the entire protein (Fig. 3*B*), disabling us to generate a hypothesis for a single region conferring the species differences. Therefore, we followed a hypothesis-free strategy by generating and analyzing a series of human–mouse chimeric OCT1 proteins. First, we generated low-resolution chimeric constructs by separating the OCT1 protein into three parts: from the N terminus to the large intracellular loop localized after transmembrane helix (TMH) 6, from TMH7 to TMH9, and from TMH10 to the C terminus (Fig. 4*A*). Comparing the concentration-dependent measurements of fenoterol and trospium uptake, the substrates with the strongest differences in the uptake between mouse and human OCT1, we identified the region from N terminus to the large intracellular loop (the first six helices of OCT1) as determining for the differences both in fenoterol and in trospium uptake (Fig. 4, *B* and *C*).

To narrow down the structures conferring the differences in uptake, we generated high-resolution chimeric constructs that carried substitutions of single TMHs of each of the first six TMHs, the large extracellular loop, and the large intracellular loop (Fig. 4*D*). The uptake by these single TMH chimeric constructs was analyzed at single concentrations of fenoterol and trospium (50 μM fenoterol and 200 μM trospium). These concentrations were chosen as they showed strong differences between wildtype human and mouse OCT1 in the concentration-dependent analyses before (Fig. 2). Introduction of mouse TMH1 into human OCT1 was the only chimera that significantly increased fenoterol uptake compared with the human wildtype (25%, *p* = 7.9 × 10^−5^; Fig. 4*E*). On the other hand, introduction of mouse TMH1 into human OCT1 (next to the introduction of TMH5) was the only one that significantly decreased trospium uptake to levels comparable or even lower than mouse Oct1 (by 91%, *p* = 4.1 × 10^−12^; Fig. 4*F*). This points to TMH1 as a major determinant of the differences in uptake between human and mouse OCT1, and we, therefore, focused our further analyses on TMH1.

### Identification of cysteine at codon 36 to tyrosine in TMH1 as the major determinant of the differences in fenoterol uptake

**Figure 5:**
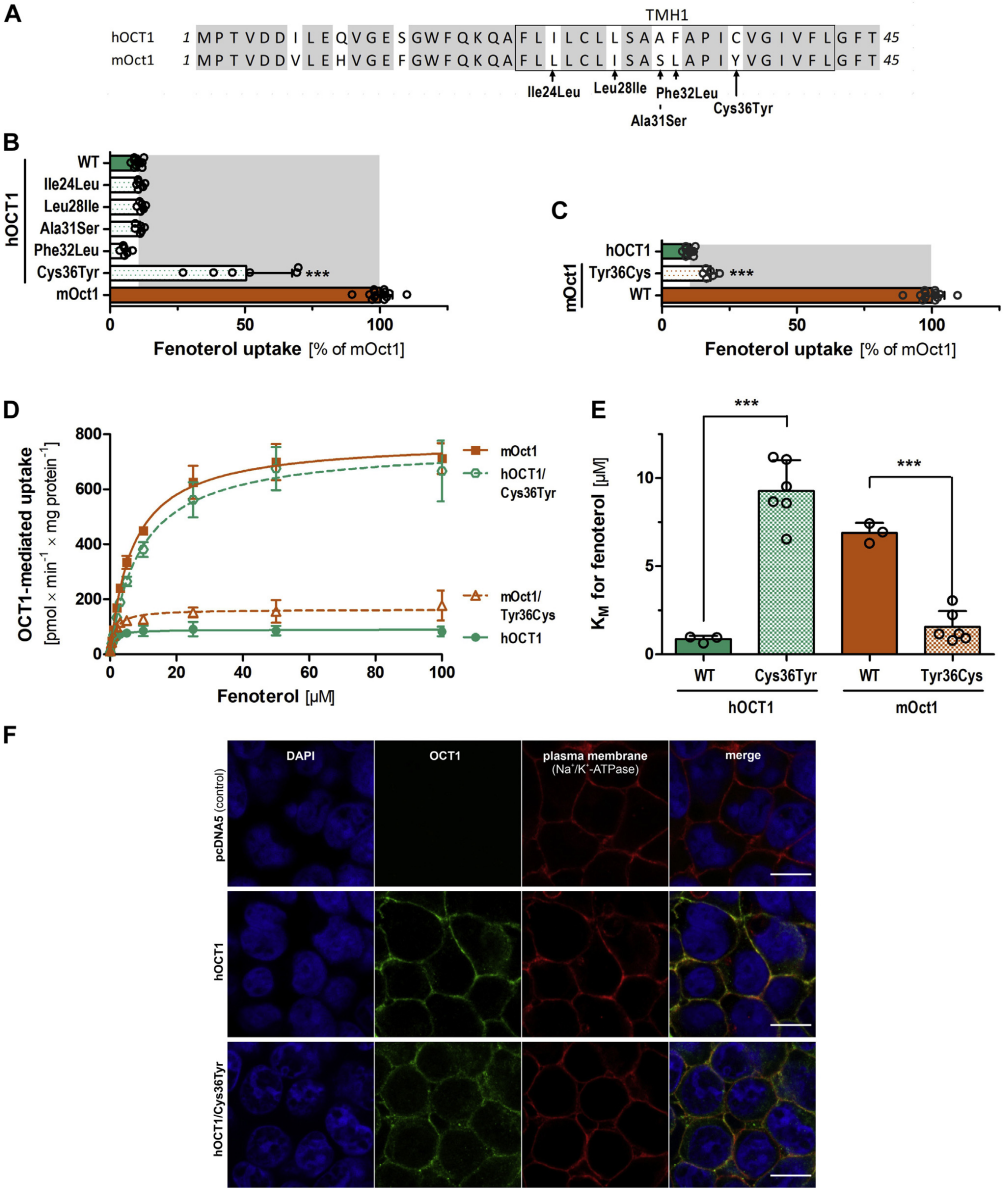
**The role of TMH1 in fenoterol transport kinetics.***A*, protein alignment of N terminus and TMH1 of human and mouse OCT1 with amino acid differences highlighted and named. *B* and *C*, effect of single substitutions of the amino acids that differ between human and mouse OCT1 on the uptake of 50 μM fenoterol. *D* and *E*, effect of substituting codon 36:Cys36Tyr in human and Tyr36Cys in mouse OCT1 on fenoterol uptake kinetics. HEK293 cells transiently (*B* and *C*) or stably (*D* and *E*) overexpressing human (*green*) and mouse (*red*) wildtype and mutant OCT1 were incubated with 50 μM (*B* and *C*) or increasing concentrations (*D* and *E*) of fenoterol for 2 min. OCT1-mediated uptake was calculated by subtracting the uptake of control cells (pcDNA5) from the uptake of OCT1-overexpressing cells. Shown are the absolute *K*_*M*_ values for fenoterol of the data shown in *D*. *E*, shown are means and standard deviation of at least three independent experiments. ∗∗∗*p* < 0.001 compared with wildtype in a Tukey’s post hoc analysis following one-way ANOVA. Membrane localization of OCT1 as assessed by immunofluorescence staining. *F*, cells were costained with an antibody against Na^+^/K^+^-ATPase as marker for the plasma membrane. Cell nuclei were stained with DAPI. The scale bar represents 10 μm. DAPI, 4′,6-diamidino-2-phenylindole; HEK293, human embryonic kidney 293 cell line; OCT1, organic cation transporter 1; TMH1, transmembrane helix 1.

In TMH1, five amino acids differ between human and mouse OCT1 (Fig. 5*A*). We substituted each of these amino acids separately with the corresponding amino acid of the other species both in human and mouse OCT1 and analyzed fenoterol uptake. The substitution of cysteine at codon 36 to tyrosine (Cys36Tyr) increased fenoterol uptake of human OCT1 by 4.8-fold, which corresponds to an increase from 10.5 to 49.8% when related to the uptake of the mouse ortholog (*p* = 2.7 × 10^−9^; Fig. 5*B*). The reverse substitution—Tyr36Cys—in mouse Oct1 reduced fenoterol uptake by 90%, resulting in uptake levels comparable to human OCT1 (*p* = 7.2 × 10^−13^; Fig. 5*C*). Mutation of the other amino acids in TMH1 or the N terminus did not affect fenoterol uptake (Figs. 5*B* and S3).

Furthermore, we generated stably transfected HEK293 cells that overexpress Cys36Tyr on human OCT1 background and Tyr36Cys on mouse Oct1 background (Fig. 5, *D* and *E*). Concentration-dependent measurements demonstrate that this single amino acid substitution was sufficient to completely reverse the uptake kinetics of fenoterol, both in terms of maximal velocity (*v*_max_, Fig. 5*D*) and affinity (*K*_*M*_, Fig. 5*E*). This confirmed the amino acid difference at codon 36—Cys36 in human and Tyr36 in mouse OCT1—as sufficient to confer the differences in fenoterol uptake.

### Identification of fenoterol moieties involved in the species-specific interactions with Cys36

After identifying Cys36 as the amino acid that is responsible for the high affinity of human OCT1, we aimed to identify moieties within the other interaction partner, the ligand fenoterol.

**Figure 6:**
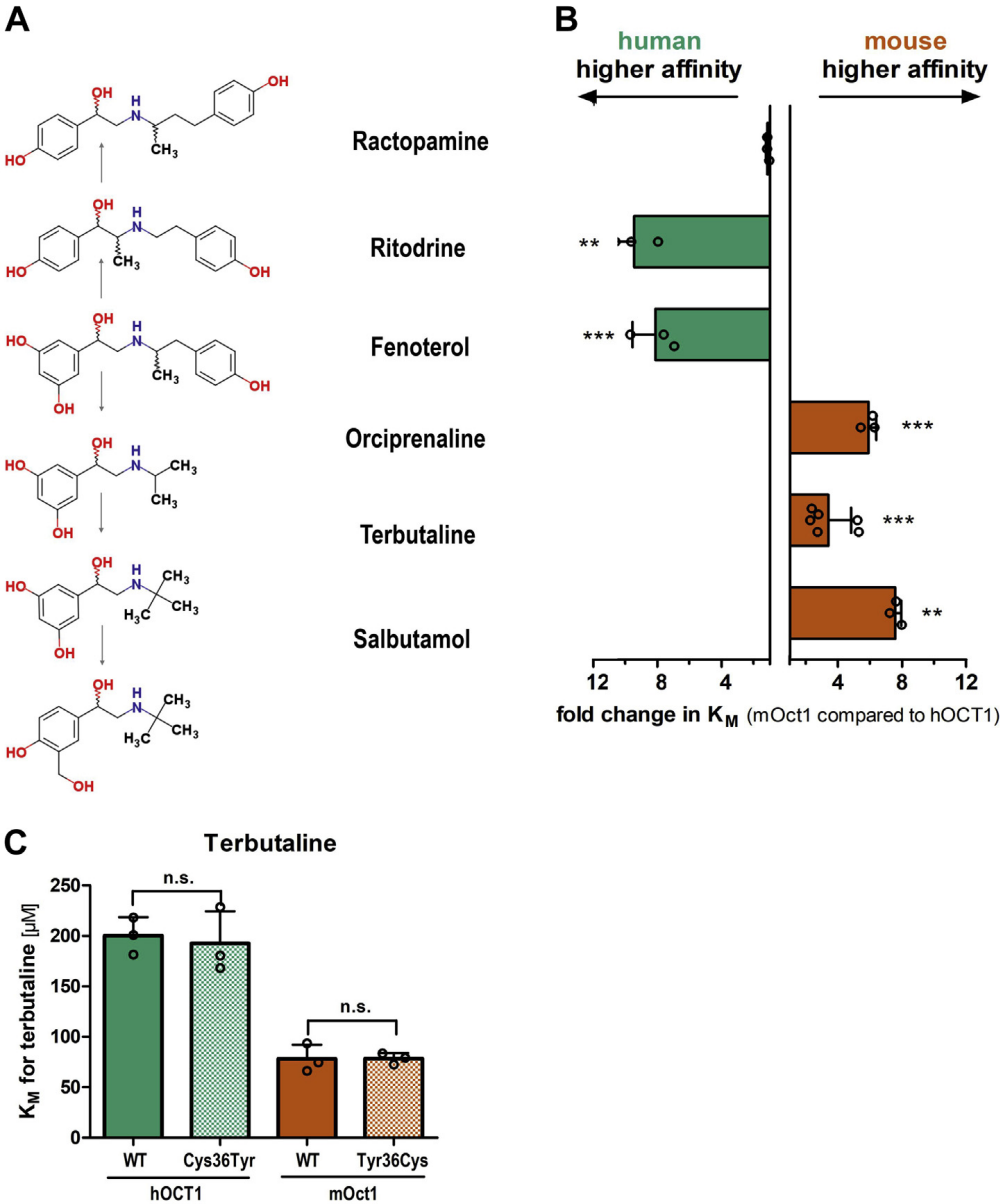
**Ligand structure walking: differences in affinity for β2-adrenergics between human and mouse OCT1.** Chemical structures of β2-adrenergics analyzed (*A*). HEK293 cells stably overexpressing human (*green*) and mouse (*red*) OCT1 (*B*) or Cys36Tyr and Tyr36Cys substituted OCT1 (*C*) were incubated with increasing concentrations of β2-adrenergics for 2 min. OCT1-mediated uptake was calculated by subtracting the uptake of control cells (pcDNA5) from the uptake of OCT1-overexpressing cells. Shown is the relative fold change in *K*_*M*_. Shown are means and standard deviation of at least three independent experiments. ∗*p* < 0.05, ∗∗*p* < 0.01, and ∗∗∗*p* < 0.001 in a Student’s *t* test with Bonferroni correction (*B*) or in a Tukey’s post hoc analysis following one-way ANOVA compared with hOCT1 (∗) or mOct1 (+) (*C*). HEK293, human embryonic kidney 293 cell line; hOCT1, human OCT1; mOct1, mouse OCT1; OCT1, organic cation transporter 1.

To this end, we compared the uptake of fenoterol and other structurally similar compounds of the class of β2-adrenergics between human and mouse OCT1. Fenoterol is composed of two phenol rings connected by a linker containing an amino group, a hydroxyl group, and a methyl group. Ritodrine is structurally the most similar, differing only in the number of hydroxyl groups and the position of the methyl group, whereas for ractopamine, the linker is composed of one additional carbon, changing the distance between the two phenol rings (Fig. 6*A*). Terbutaline, orciprenaline, and salbutamol share many features with fenoterol but lack the second phenol ring and vary in the number and positions of their methyl and hydroxyl groups.

Comparing the transport affinity between human and mouse OCT1, ritodrine with the most similar structure also showed similar transport kinetics to fenoterol, with an almost 10-fold higher affinity of human OCT1 (Fig. 6*B*). In contrast, the absence of the second phenol ring such as in terbutaline, orciprenaline, and salbutamol led to a switch in transport affinity to a more than sixfold higher affinity of mouse Oct1. Interestingly, despite the similar structure, species-specific effects differ between fenoterol and ractopamine. In contrast to fenoterol, affinity for ractopamine was comparable between human and mouse OCT1 (Fig. 6*B*). This indicates that the presence of two phenol rings such as in fenoterol and ritodrine is not sufficient for higher affinity to the human OCT1, but the distance of the second aromatic ring to the positive charge of the protonable amino group may be relevant. Also the substrate-specific differences in the uptake of formoterol, adrenaline, and noradrenaline reported previously (Fig. 1 and Table 1) support these findings.

In line with this, the substitutions Cys36Tyr in human and Tyr36Cys in mouse OCT1 did not affect the affinity for terbutaline (Fig. 6*C*). This, taken together with the significant effects of these substitutions on fenoterol uptake (Fig. 5*E*), further supports a direct interaction of Cys36 with the second phenol ring of fenoterol.

**Figure 7:**
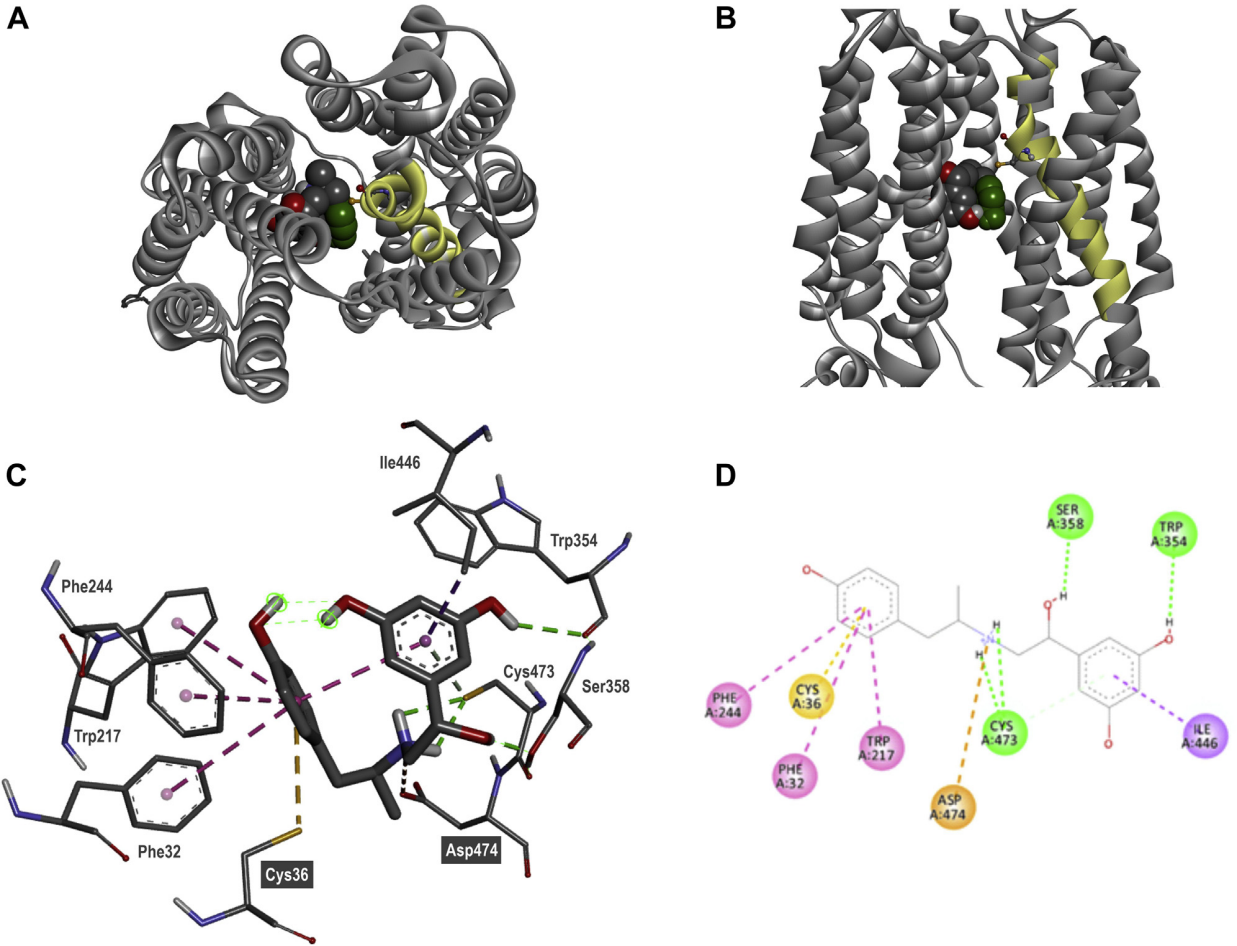
**Docking pose showing the interaction of fenoterol with human OCT1.** Fenoterol (S,S enantiomer) was docked into the protein structure model of human OCT1 from AlphaFold DB (UniProt ID: O15245) using AutoDock 4.2.6. *Top* (*A*) and *side* (*B*) view of fenoterol docking into human OCT1 with TMH1 highlighted in *yellow*. Detailed 3D (*C*) and 2D (*D*) representation of intermolecular interactions. Interacting amino acids are labeled, the type of interaction is color coded: *pink*, pi–pi T shaped; *yellow*, pi–sulfur; *orange*, attractive charge; *green*, hydrogen bond; and *violet*, pi–sigma. OCT1, organic cation transporter 1; TMH1, transmembrane helix 1.

Next, we took advantage of an artificial intelligence–generated high-accuracy protein structure model of human OCT1 available from the AlphaFold Protein Structure Database. Molecular docking allowed us to study *in silico* the possible interactions between fenoterol, Cys36, and other residues in human OCT1. Pose clustering allowed us to identify meaningful interactions by considering with higher priority poses with reoccurring interaction pattern within a cluster (here: cluster 4; Fig. S7), which were at the same time ranked high in terms of approximated binding energies. Fenoterol seems to be stabilized *via* a network of interactions with human OCT1, which leads to a U-shaped conformation of fenoterol (additionally stabilized by an intramolecular H-bond formed by two OH groups of the oppositely located phenolic rings in fenoterol; Figure 7). In the linker part connecting the two aromatic rings of fenoterol, the positively charged nitrogen is forming an ionic interaction with Asp474, and Cys473 is forming H-bonds with the NH_2_ group. In addition, the Ser358 side chain forms an H-bond with the OH group of the linker. The aromatic ring possessing two OH groups in fenoterol is stabilized by both a conventional H-bond with Trp354 as well as a pi–sigma interaction with Ile446. The second phenol ring seems to be trapped in a network of T-shaped pi–pi interactions with the aromatic residues of Phe32, Phe244, and Trp217. Most importantly, the crucial Cys36 interaction in addition stabilizes this ring *via* a pi–sulfur interaction.

### Identification of phenylalanine at codon 32 to leucine in TMH1 as the major determinant of the species differences in trospium transport

**Figure 8:**
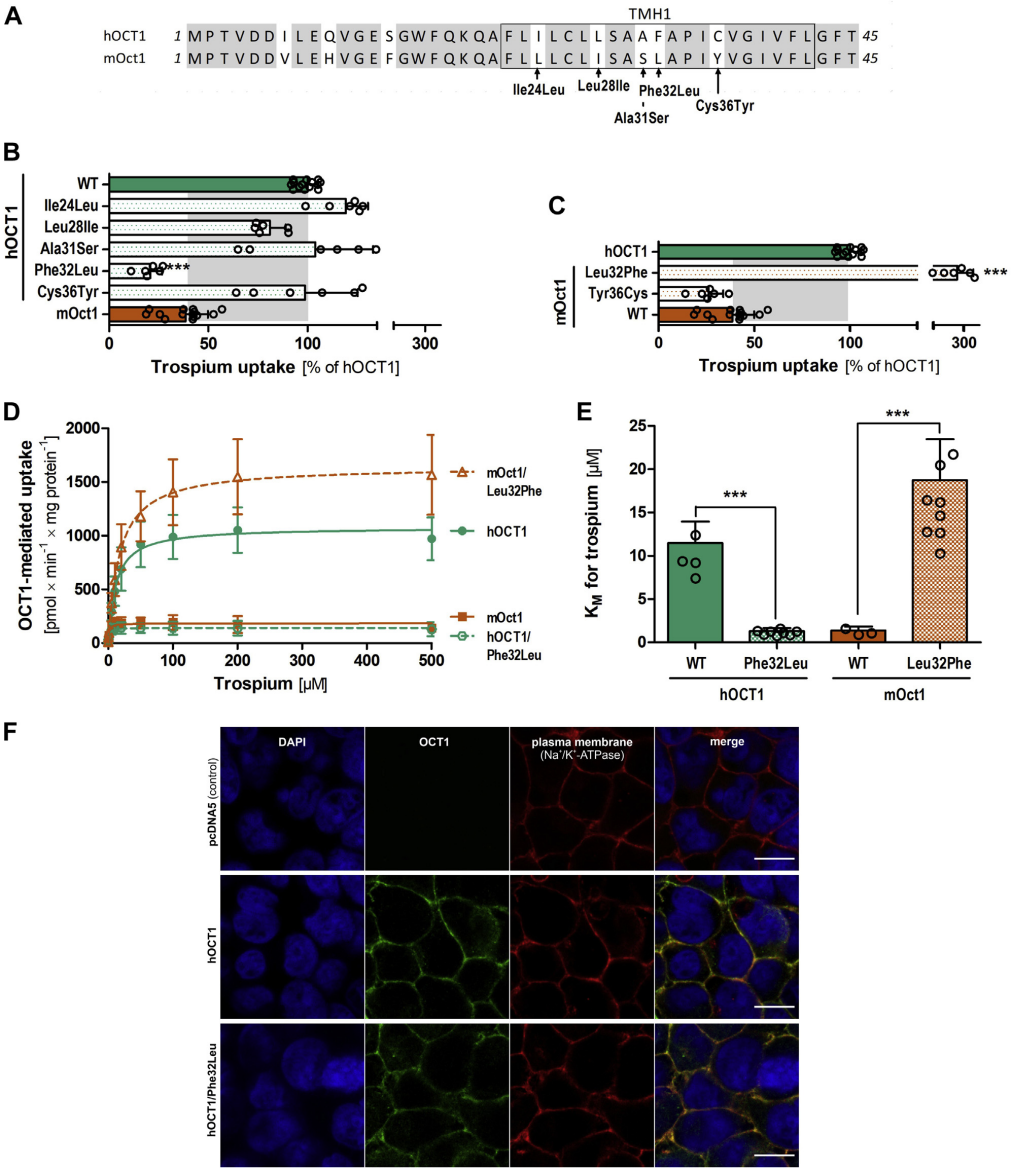
**The role of TMH1 in trospium transport kinetics.***A*, protein alignment of N terminus and TMH1 of human and mouse OCT1 with amino acid differences highlighted and named. *B* and *C*, effect of single substitutions of the amino acids that differ between human and mouse OCT1 on the uptake of 200 μM trospium. *D* and *E*, effect of substituting codon 32:Phe32Leu in human and Leu32Phe in mouse OCT1 on trospium uptake kinetics. HEK293 cells transiently (*B* and *C*) or stably (*D* and *E*) overexpressing human (*green*) and mouse (*red*) wildtype and mutant OCT1 were incubated with 200 μM (*B* and *C*) or increasing concentrations (*D* and *E*) of trospium for 2 min. OCT1-mediated uptake was calculated by subtracting the uptake of control cells (pcDNA5) from the uptake of OCT1-overexpressing cells. Shown are the absolute *K*_*M*_ values for trospium of the data shown in *D*. *E*, shown are means and standard deviation of at least three independent experiments. ∗∗∗*p* < 0.001 compared with wildtype in a Tukey’s post hoc analysis following one-way ANOVA. *F*, membrane localization of OCT1 as assessed by immunofluorescence staining. Cells were costained with an antibody against Na^+^/K^+^-ATPase as marker for the plasma membrane. Cell nuclei were stained with DAPI. The scale bar represents 10 μm. The staining of the positive (hOCT1) and negative (pcDNA5) control cells is identical to the one shown for Figure 5*F* and is shown here again to facilitate the direct comparison to the Phe32Leu mutant. DAPI, 4′,6-diamidino-2-phenylindole; HEK293, human embryonic kidney 293 cell line; hOCT1, human OCT1; OCT1, organic cation transporter 1; TMH1, transmembrane helix.

Similar to fenoterol, chimeric constructs pointed to TMH1 as determinant of the differences in trospium uptake between human and mouse OCT1 (Fig. 8, *C* and *F*). Similar to fenoterol, we analyzed the effects of single amino acid substitutions in TMH1 on trospium uptake. The substitution of phenylalanine at codon 32 to leucine (Phe32Leu) in human OCT1 decreased trospium uptake by 79% (*p* = 1.5 × 10^−10^; Fig. 8*B*) to levels even lower than the uptake levels of mouse Oct1. The reverse substitution in mouse Oct1, Leu32Phe, increased trospium uptake by sevenfold. This corresponds to levels that even exceeded the uptake of the human ortholog at the same concentrations by 2.8-fold (Fig. 8*C*). In contrast to fenoterol, Cys36Tyr did not affect uptake of trospium. Also the substitutions of any other amino acid within TMH1 or the N terminus did not affect the uptake of trospium (Figs. 8*B* and S3, *D* and *E*). Substitution of codon 32 (Phe32Leu and Leu32Phe) did not affect fenoterol uptake (Figs. 5*A* and S3, *B* and *C*), suggesting strongly substrate-specific effects of this amino acid.

We also generated stably transfected HEK293 cells that overexpress Phe32Leu on human OCT1 background and Leu32Phe on mouse Oct1 background. Concentration-dependent measurements using the stably transfected HEK293 cells confirmed the effects on *v*_max_ and *K*_*M*_ (Fig. 8, *D* and *E*). This confirmed the amino acid difference at codon 32—Phe32 in human and Leu32 in mouse OCT1—as sufficient to confer the differences in trospium uptake.

## Discussion

In this study, we report substantial differences in the substrate specificity between human and mouse OCT1 orthologs. Twenty-two of 28 substrates tested (79%) showed twofold or higher differences in OCT1 affinity and/or capacity. This finding reflects the polyspecificity of OCT1 and has direct consequences for translation of OCT1 functional data from mouse to human. Furthermore, high species-specific variability in OCT1 function can be used as a tool for identifying substrate-specific interactions of the OCT1 transporter with its ligands. Applying a hypothesis-free approach involving low-resolution and high-resolution chimeric constructs followed by site-directed mutagenesis, we were able to identify single amino acid substitutions that were sufficient to explain the two most extreme differences in affinity between human and mouse OCT1 orthologs.

Fenoterol was the substrate with the highest affinity of human OCT1 in this study (Table 1) and is among the OCT1 substrates with the highest affinity known in general (14). We were able to identify a single amino acid substitution—Cys36 (human) to Tyr36 (mouse)—that was sufficient to completely reverse the uptake kinetics of fenoterol between human and mouse and to make fenoterol a substrate of average affinity in human OCT1. Vice versa, trospium was among the substrates transported with the highest affinity by mouse Oct1 in this study. In this case, we were also able to identify a single amino acid difference between the species—Leu32 (mouse) to Phe32 (human)—whose substitution was sufficient to completely reverse the species differences in transport kinetics and to make trospium a substrate of average affinity in mouse Oct1.

To the best of our knowledge, this study delivers the first experimental evidence for an important role of amino acids in TMH1 in OCT1 substrate recognition. The majority of the functional analyses in the past were focused on TMH4 and TMH10 (21, 23). The best example is Asp474 (Asp475 in rodent Oct1) in TMH11. Asp474 is universally accepted to be essential for OCT1 cation interaction (17, 18, 20, 36, 37). The amino acids Phe32 and Cys36 were mentioned in two previous studies as potentially involved in OCT1 ligand interaction (18, 19). In both studies, the two amino acids were identified by *in silico* analyses, were not experimentally validated, and were among more than 10 other amino acids suggested to be of potential relevance.

Cys36 and Phe32 showed strongly substrate-specific effects. The Cys36Tyr substitution led to substantial differences in the affinity for fenoterol and the structurally closely related ritodrine, but did not affect the affinity for trospium or even for other β2-adrenergics like terbutaline (Figs. 6*C* and 8, *B* and *C*). Substitution of codon 32 affected trospium but not fenoterol kinetics. Interestingly, Cys36 and Phe32 are located in very close proximity in TMH1 but affect different substrates in a substrate-specific manner. This suggests that TMH1 may harbor multiple mechanisms conferring polyspecificity.

The amino acids Cys36 and Tyr36 as well as Phe32 and Leu32 show distinct conservation patterns within other mammalian OCT1 orthologs (Fig. S4). Cys36 is present only in human and primates, whereas the corresponding Tyr36 in mouse Oct1 is conserved throughout most of the mammalian orthologs analyzed. In contrast, Phe32 is conserved throughout most mammalian orthologs, except for rodents. Mouse, rat, hamster, prairie vole, and chinchilla Oct1 have Leu32.

Substrate-specific effects of amino acids in OCT1 have been reported before but only for model substrates like MPP^+^ and TEA^+^ (38). Today, technological advancements of analytical methods such as LC–MS/MS enable the analysis of practically any substance of interest. This will help to extend mutagenesis experiments beyond the conventionally used model substrates and thereby help to better understand the polyspecificity of OCT1 with a focus on clinically more relevant substrates like drugs or endogenous metabolites.

The strong differences in uptake kinetics for fenoterol and trospium observed between human and mouse OCT1 *in vitro* are not expected to lead to substantial differences in the pharmacokinetics in the two species. The reason for this is that the effects of affinity and transport capacity neutralize each other at clinically relevant concentrations. Despite a more than eightfold difference in transport affinity (*K*_*M*_), differences in transport capacity (*v*_max_) in the opposite direction lead to almost identical OCT1 intrinsic clearances for both OCT1 orthologs (Table 1). Indeed, when comparing the uptake of human and mouse OCT1 at low concentrations (100 nM), we did not see differences in uptake, neither for fenoterol nor for trospium (Fig. S5). These experimentally tested concentrations are still above the clinically relevant ones (2.18 nM for fenoterol (9) and 13.3 nM for trospium (39)), but extrapolation based on intrinsic clearances suggests that the lack of species differences in the uptake will sustain also at lower concentrations. One direct consequence of this observation is that data about the effects of Oct1 on the hepatic uptake of fenoterol in mice (12) may closely reflect OCT1 effects in humans.

In contrast to fenoterol and trospium, strong differences in the intrinsic clearance between human and mouse OCT1 were observed for 11 other substrates (Fig. S6). Two of them—the antidiabetic metformin and thiamine (vitamin B1)—were reported by us before (34). In this work, the number of substances that are expected to have clinically relevant species differences in the OCT1-mediated clearances was extended to the β2-adrenergic drugs formoterol, terbutaline, and salbutamol, the model substrate TEA^+^ (which is probably of theoretical interest only as TEA^+^ could not be administered to humans), and a number of endogenous substances.

The much better uptake of endogenous substrates by mouse Oct1 may reflect evolutionary adaptation and different physiological roles of OCT1 in humans and mice. For the majority of the endogenous substrates tested, mouse Oct1 had a higher intrinsic clearance (Fig. 1 and Table 1), suggesting stronger hepatic uptake or uptake by other OCT1-expressing organs. The remaining endogenous compounds such as IBC and choline were not transported at all by human OCT1 but showed strong uptake by mouse Oct1. Differences in substrate spectra between species have been reported for other pharmacologically relevant transporters such as multidrug resistance–associated protein 2 (MRP2) and for several cytochrome P450 enzymes (40, 41, 42, 43, 44). Our data also suggest that especially effects of OCT1 on physiological processes, for example, obtained by analyzing Oct1 knockout mice, should be carefully interpreted in terms of translation to humans.

Our study demonstrates that next to the “classical” machine learning approaches, where large datasets of ligands with substantially different structures are analyzed (18, 45, 46), also detailed analyses of ligands with very similar chemical structures may be applied to identify single moieties involved in the interaction with OCT1. Here, we applied analyses within the group of β2-adrenergic drugs sorting them by single structural substitutions (Fig. 6*A*)—an approach we call “ligand structure walking.” Analyzing the species-specific differences in the uptake for each step of β2-adrenergics structure changes, we were able to point to the second phenol ring as the major ligand structure determinant of the interaction with Cys36 and thus determining the high affinity of human OCT1 for fenoterol. Furthermore, “walking” further down on the structures of β2-adrenergics, we observed that the affinity of human OCT1 for ractopamine is not much higher than the one of mouse Oct1, despite ractopamine also possessing a second phenol ring. This may be explained by the larger distance between the second phenol ring of ractopamine and the positively charged amino group compared with the distance in fenoterol. This suggests that not only the presence of a second ring but also the distance between the second ring and the protonable amino group may play an important role in the high affinity for fenoterol.

The experimentally identified interaction between the second phenol ring of fenoterol and Cys36 in human OCT1 could also be confirmed using molecular docking (Fig. 7). However, we should acknowledge that the interaction was observed in only four of the 35 inspected poses belonging to the five highest ranked clusters of docking poses (Fig. S7), and none of them was among the 10 energetically most favorable ones. Thus, the computational models cannot inconclusively describe the essentiality of Cys36 for the high-affinity interaction of fenoterol with human OCT1 to date. In the future, molecular dynamics simulations may help to shed more light on the energetically most favorable interactions.

On the other hand, molecular docking suggested that in addition to Cys36, Phe32 also interacts with the second ring of fenoterol (Fig. 7). However, our experimental data cannot support this. Substitution of Phe32Leu did not affect fenoterol uptake (Fig. 5), although Leu32 cannot be involved in a pi–pi interaction, as suggested for Phe32 (Fig. S7*B*). Another amino acid suggested by *in silico* docking to interact with the second ring of fenoterol is Phe244. This was also suggested in a recent publication by Gebauer *et al.* (47) but for the (S,S) enantiomer of fenoterol only.

In conclusion, here we report substantial differences in the uptake kinetics between human and mouse OCT1. Endogenous substrates and multiple β2-adrenergic drugs were transported with much higher intrinsic clearance by mouse than by human OCT1, warranting caution when translating pharmacokinetics and physiological data about OCT1 from mice to humans. More importantly, we were able to identify single amino acids that are strongly involved in substrate-specific interaction of OCT1. Cys36 conferred the extremely high affinity of human OCT1 for fenoterol in a highly substrate-specific manner, and Leu32 conferred the high affinity of mouse Oct1 for trospium. These previously unknown amino acids important for OCT1 polyspecificity were identified by using a hypothesis-free comparison of human and mouse OCT1 orthologs. This is another step in understanding the mechanisms of OCT1 polyspecificity and describes an approach that is extendable to other OCT1 orthologs and paralogs.

## Experimental procedures

### Reagents

Ipratropium bromide, IBC, IBC-d6, l-noradrenaline, ritodrine hydrochloride, sumatriptan-d6, thiamine-d3 hydrochloride, and trospium chloride were obtained from Santa Cruz Biotechnology. Ranitidine-d6 and trospium-d8 were obtained from Toronto Research Chemicals, ASP^+^ was obtained from Life Technologies, and buformin hydrochloride was obtained from Wako Chemicals. Radiolabeled MPP^+^ (*N*-[methyl-^3^H], 80 Ci/mmol) and TEA^+^ ([ethyl-1-^14^C]; 55 mCi/mol) were obtained from Hartmann Analytic. All other compounds tested as substrates were obtained from Sigma–Aldrich. All chemicals used in this study were purchased from commercial sources and had purities of 97% or higher.

Dulbecco's modified Eagle's medium, Hank's buffered salt solution (HBSS), and additives used for cell culturing were obtained from Life Technologies. Poly-d-lysine (1–5 kDa), Hepes, bicinchoninic acid, and copper sulfate pentahydrate were obtained from Sigma–Aldrich. Twelve-well plates were obtained from Starlab, and tissue culture flasks were from Sarstedt. Acetonitrile and methanol in LC–MS/MS grade were obtained from LGC Standards, and formic acid (LC–MS/MS grade) and sodium chloride were obtained from Merck. SDS (UltraPure) was obtained from AppliChem.

### Cell lines and cell culturing

HEK293 cells stably overexpressing human OCT1, mouse Oct1, human–mouse chimeric OCT1, or human and mouse mutant OCT1 were generated by targeted chromosomal integration using the Flp-In System (Life Technologies). Generation and characterization of these cell lines has been described in detail before (34, 48, 49). Cells were cultured in Dulbecco’s Modified Eagle’s Medium supplemented with 10% fetal bovine serum, 100 U/ml penicillin, and 100 μg/ml streptomycin at 37 °C and 5% CO_2_ and were passaged twice a week.

### Generation of OCT1 expression constructs

For overexpression of OCT1 in HEK293 cells, pcDNA5/FRT expression vectors (Thermo Fisher Scientific) containing wildtype, chimeric, or mutant human or mouse OCT1 constructs were generated as described previously (34, 48, 49). Human–mouse chimeric OCT1 constructs were generated using the overlap extension method (50) and have been characterized before (34). Point mutations were introduced into pcDNA5/FRT vectors containing human or mouse OCT1 by site-directed mutagenesis using the primers listed in Table S2. All constructs were validated by capillary sequencing of the complete open reading frame of OCT1 before transfection into HEK293 cells.

### Transient transfection of T-REx-293 cells for cellular uptake experiments

For transient transfection of OCT1 constructs into HEK293 cells for uptake experiments, 5 × 10^5^ T-REx-293 cells (Life Technologies) were seeded per well in 12-well plates precoated with poly-d-lysine. At 24 h later, cells were transfected with 100 μl of reaction mix per well, containing 2 μg pcDNA5/FRT vector with the OCT1 construct of interest, 0.5 μg pGFP-tpz vector, and 6.25 μl Lipofectamine 2000 (Thermo Fisher Scientific) according to the manufacturer’s instructions. At 48 h later, transfection efficacy was assessed microscopically by visualizing the GFP signal of the cotransfected GFP vector, and the cells were subsequently used for uptake experiments.

### Cellular uptake experiments

At 48 h prior to the experiment, 6 × 10^5^ cells were seeded per well in 12-well plates. When using transiently transfected cells, 5 × 10^5^ T-REx-293 cells (Life Technologies) were seeded per well in 12-well plates 72 h prior to the experiment, and they were transfected 24 h later as described previously. Twelve-well plates were precoated with poly-d-lysine.

Cellular uptake experiments were performed at 37 °C and pH 7.4 using HBSS supplemented with 10 mM Hepes (in the following referred to as HBSS+). Cells were washed with 1 ml prewarmed (37 °C) HBSS+, and the uptake was initiated by adding 400 μl of prewarmed HBSS+ containing the substrate. Uptake was allowed for exactly 2 min and afterward stopped by adding 2 ml ice-cold HBSS+. Cells were washed twice with 2 ml ice-cold HBSS+ and lysed with 80% acetonitrile supplemented with internal standard, 0.1 N NaOH/0.1% SDS, or radioimmunoprecipitation buffer for LC–MS/MS, liquid scintillation counting, or fluorescence spectroscopy detection, respectively. Intracellular substrate concentrations were measured as described in the following and were then normalized to the amount of total protein in the samples as measured using the bicinchoninic acid assay (51).

### Quantification of intracellular substrate concentration by LC–MS/MS

For LC–MS/MS quantification of intracellular substrate concentrations, the cell lysate was centrifuged at 16,000*g* for 15 min, and 350 μl of supernatant was evaporated to dryness under nitrogen flow at 40 °C. The sample was reconstituted with 200 μl 0.1% formic acid, and between 2 and 30 μl were injected into the LC–MS/MS system (Table S1).

An API4000 QTRAP tandem mass spectrometer with ESI interface (AB SCIEX) was used, coupled to a Shimadzu Nexera X2 UHPLC system with LC 30AD pumps and SiL 30AC autosampler (Shimadzu). Samples were separated on a Brownlee SPP RP-Amide column (4.6 × 100 mm, 2.7 μm; PerkinElmer) using a mobile phase of 0.1% (v/v) formic acid and varying concentrations of organic solvent (Table S1).

### Quantification of intracellular substrate concentration by liquid scintillation counting

Intracellular concentrations of radioactively labeled substrates MPP^+^ and TEA^+^ were quantified using liquid scintillation counting. To this end, 400 μl cell lysate was transferred into a 20 ml scintillation vial, 9 ml Aquasafe 500 Plus liquid scintillator (both Zinsser Analytics) was added, and the sample was measured in the Scintillation Counter LS6500 (Beckman Coulter).

### Quantification of intracellular substrate concentration by fluorescence spectroscopy

For fluorescence spectroscopy detection of intracellular ASP^+^ concentrations, 200 μl cell lysate was transferred into a black 96-well plate, and fluorescence was measured in a Tecan Ultra Microplate Reader (Tecan Group) with excitation and emission wavelengths of 485 and 612 nm, respectively.

### Immunocytochemical staining and confocal microscopy analysis of OCT1-overexpressing cells

For immunocytochemical staining of OCT1, 6 × 10^5^ HEK293 cells stably overexpressing mutant or wildtype OCT1 were seeded onto cover slips in 12-well plates precoated with poly-d-lysine 48 h prior to staining. Cells were washed twice with 1 ml Dulbecco's PBS (DPBS) for 10 min and fixed with 100% ethanol for 20 min at −20 °C. After washing three times with DPBS for 5 min, cell membranes were permeabilized with DPBS/0.4% Tween-20 for 10 min. Cells were washed three times with DPBS for 5 min and blocked with blocking buffer (DPBS/5% fetal bovine serum) for 1 to 2 h. Cells were incubated with the primary antibodies diluted in blocking buffer in a humid chamber overnight at 4 °C. Monoclonal antibodies mouse anti-OCT1 2C5 (NBP1-51684; Novus Biologicals) and rabbit anti-Na^+^/K^+^-ATPase (EP1845Y; Abcam) were diluted 1:400 and 1:200, respectively. The next day, after washing three times with DPBS for 5 min, cells were incubated with the secondary antibodies diluted in blocking buffer for 1 to 2 h protected from light. Polyclonal antibodies Alexa Fluor 488 goat antimouse immunoglobulin G (H + L) and Alexa Fluor 568 goat anti-rabbit immunoglobulin G (H + L; both Thermo Fisher Scientific) were diluted 1:400. After washing three times with DPBS for 5 min, cover slips were mounted onto microscope slides using ROTI mount FluorCare 4′,6-diamidino-2-phenylindole (Carl Roth). Cells were analyzed using a laser scanning microscope (LSM780; Carl Zeiss) with ZEN 2010 software, version 6.0, and images were processed using the Fiji distribution of ImageJ2 (52, 53).

### Computational modeling

The AlphaFold version 2.0 AI system of DeepMind (54) has enabled the prediction of highly accurate protein models, which are made available *via* the openly accessible AlphaFold Protein Structure Database (AlphaFold DB (55); https://alphafold.ebi.ac.uk/). The structural model of human OCT1 (UniProt ID: O15245) was downloaded in Protein Data Bank format from the AlphaFold DB and used for molecular docking studies using AutoDock 4.2.6 (56). The chemical structure of (S,S)-fenoterol was downloaded from PubChem (https://pubchem.ncbi.nlm.nih.gov/) in SDF format (Pubchem CID: 3343), and the protonation state at pH 7.4 was calculated in MarvinSketch, version 20.18 (https://chemaxon.com/products/marvin). Explicit hydrogen atoms were added to the ligand accordingly. Protein preparation was performed in AutoDock Tools (https://autodock.scripps.edu/). Specifically, polar hydrogens and charges were added, and the structure was saved in PDBQT format. Next, a grid parameter file was generated describing the binding site of the protein, guided by the identified site in our previous work (34). AutoDock uses a Lamarckian Genetic Algorithm and an empirical binding free energy function for molecular docking (56). The number of poses was set to 100 and a population size of 300 for better sampling of the 3D space (other parameters were set to default).

The docking output also provides a clustering histogram containing information about the predicted binding energies of the docked structures and the similarities of the docked conformations to each other (by RMSD). AutoDock clusters the docked results at 0.5 Å by default, ordering the conformations by docked energy, from lowest to highest. Poses were visually inspected in Discovery Studio Visualizer (https://discover.3ds.com/discovery-studio-visualizer-download). Docking poses are also summarized in a 2D diagram, visualizing all different types of interactions in a color-coded manner. Criteria for selection of a representative pose included the selection based on low approximated binding energy, frequency of the observed interactions, as well as membership to a highly populated cluster and interaction with Asp474.

### Data analyses

The kinetic transport parameters *K*_*M*_ and *v*_max_ were determined by nonlinear regression to the Michaelis–Menten equation using GraphPad Prism, version 5.01 (GraphPad Software, Inc). Kinetic parameters between human and mouse OCT1 were compared using the Student’s *t* test with Bonferroni correction. Kinetic parameters or uptake values between three or more groups, that is, human and mouse OCT1 and human–mouse chimeric OCT1 or mutant OCT1 were compared using ANOVA followed by Tukey’s honestly significant difference post hoc comparisons. For both analyses, SPSS Statistics, version 28 (SPSS, Inc, IBM) was used.

## Data availability

All data described are contained within this article and supporting information.

## Supporting information

This article contains supporting information.

## Conflict of interest

The authors declare that they have no conflicts of interest with the contents of this article.

## Supporting information

Supplemental Figures S1–S7 and Tables S1–S2

AF_hOCT1_Fenoterol_Cluster4_Pose80 - Kopie.pdb
