# Supplementary material for: Amino acids in transmembrane helix 1 confer major functional differences between human and mouse orthologs of the polyspecific membrane transporter OCT1
Source: J Biol Chem. 2022 Apr 22;298(6):101974. doi: 10.1016/j.jbc.2022.101974 (PMC9130538; doi:10.1016/j.jbc.2022.101974)
Supplement: Supplemental Figures S1–S7 and Tables S1–S2 [file mmc1.docx]

# Supporting information

**Amino acids in transmembrane helix 1 confer major functional differences between human and mouse orthologs of the polyspecific membrane transporter OCT1**

Marleen J. Meyer^1^, Pascale C. F. Schreier^1^, Mert Basaran^3^, Stefaniia Vlasova^3^, Tina Seitz^2^, Jürgen Brockmöller^2^, Barbara Zdrazil^3^, Mladen V. Tzvetkov^1^*

1 Department of General Pharmacology, Institute of Pharmacology, Center of Drug Absorption and Transport (C_DAT), University Medicine Greifswald, Greifswald, Germany

2 Institute of Clinical Pharmacology, University Medical Center Göttingen, Göttingen, Germany

3 Department of Pharmaceutical Sciences, Division of Pharmaceutical Chemistry, University of Vienna, Vienna, Austria

**Table of contents**

**Fig. S1S-2**

**Fig. S2S-3**

**Fig. S3S-4**

**Fig. S4S-5**

**Fig. S5S-5**

**Fig. S6S-6**

**Fig. S7S-7**

**Table S1S-8**

**Table S2S-10**


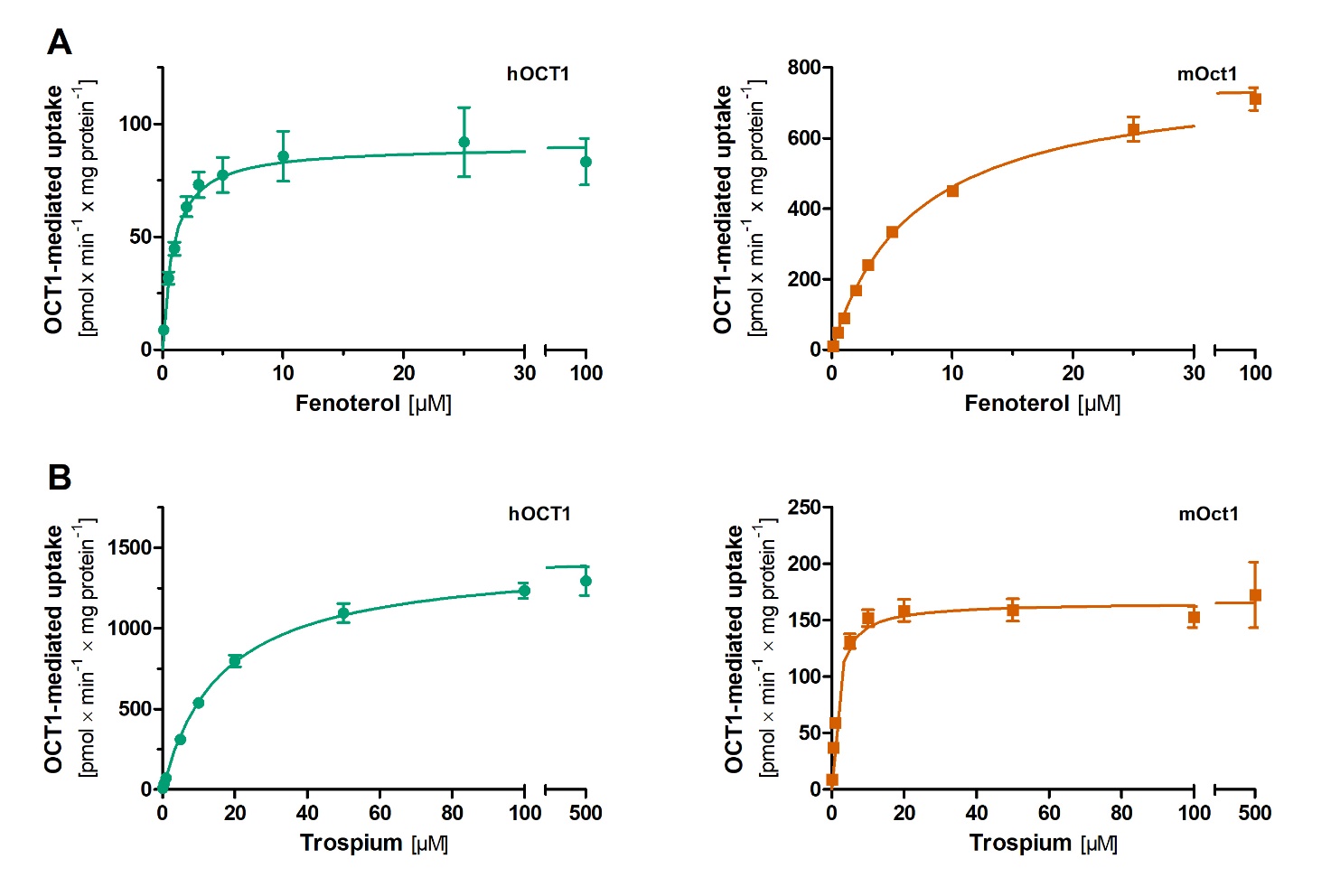


Fig. S1. **Comparative characterization of fenoterol and trospium uptake between human and mouse OCT1** **orthologs** HEK293 cells stably transfected to overexpress human (green) and mouse (red) OCT1 were incubated for 2 min with increasing concentrations of fenoterol (A) or trospium (B). OCT1-mediated uptake was calculated by subtracting the uptake of control cells (pcDNA5) from the uptake of OCT1-overexpressing cells. Shown are means and standard errors of the means of at least three independent experiments. Note that the y-axes have different ranges for human and mouse OCT1 in A and B, respectively.


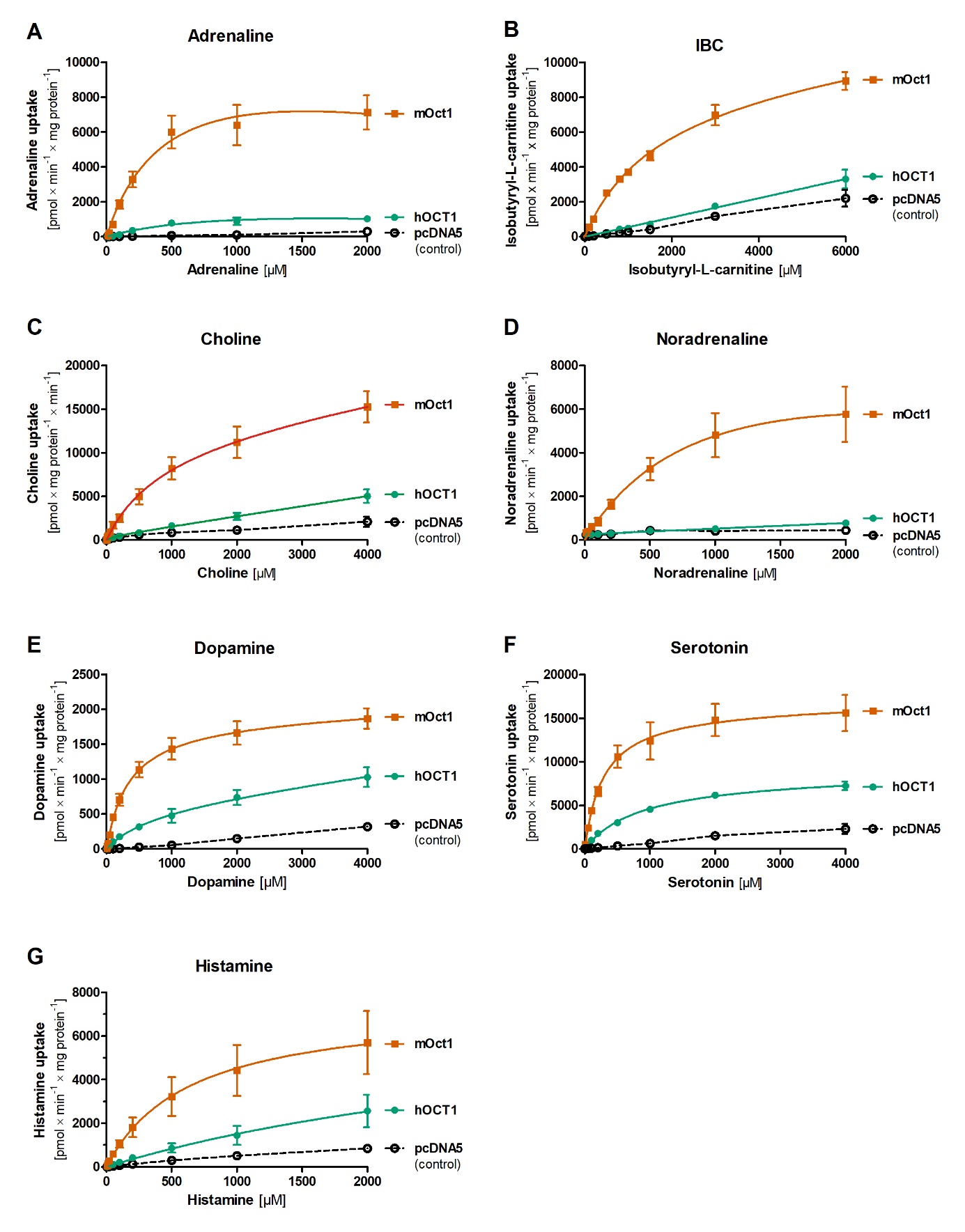


Fig. S2. **Concentration-dependent uptake of endogenous compounds by human and mouse OCT1** HEK293 cells stably overexpressing human (green) and mouse (red) OCT1 and control cells transfected with the empty vector (pcDNA5) were incubated with increasing concentrations of (A) adrenaline, (B) isobutyryl-L-carnitine (IBC), (C) choline, (D) noradrenaline, (E) dopamine, (F) serotonin, or (G) histamine for 2 min. Shown are means and standard errors of the means of at least three independent experiments.


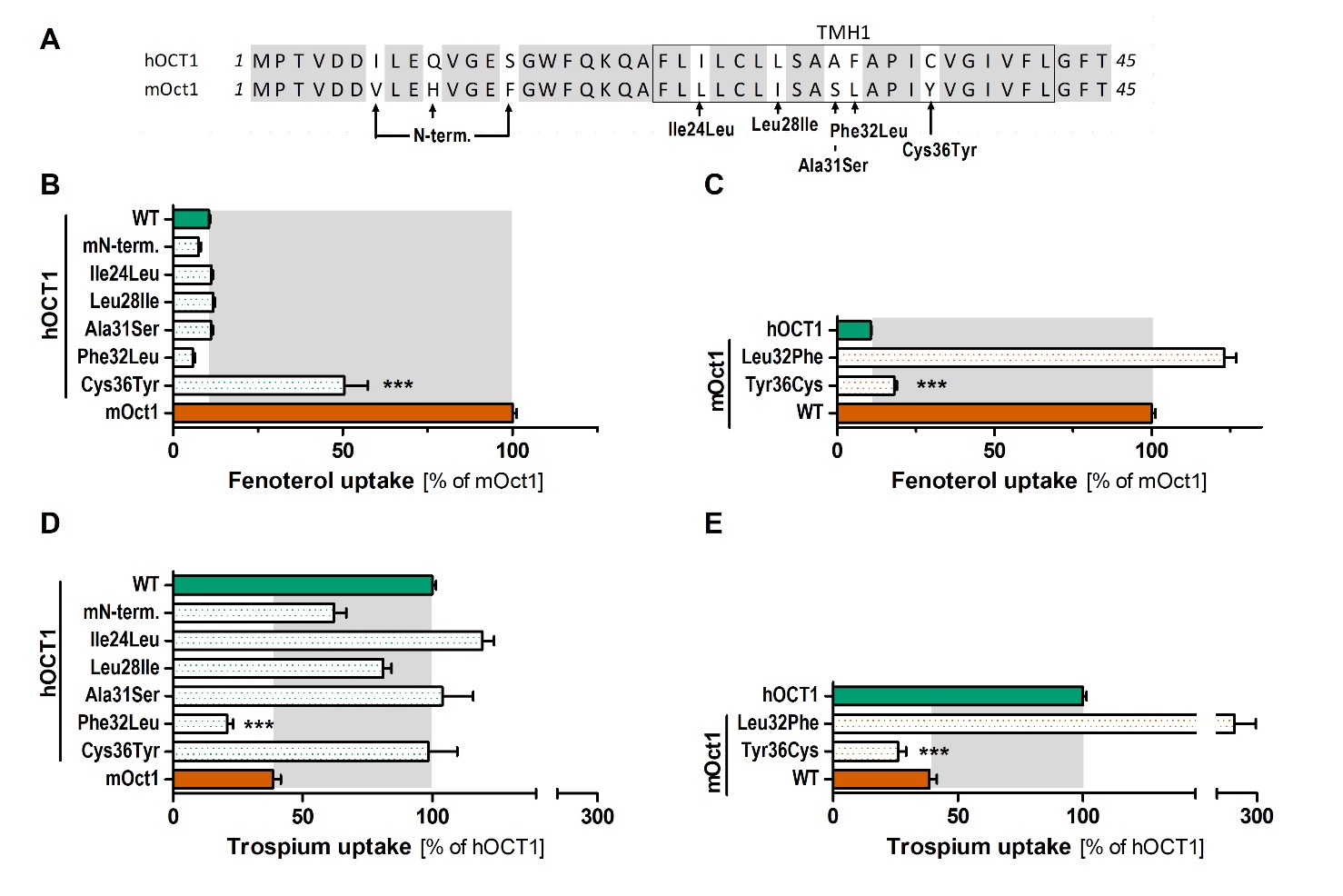


Fig. S3. **The role of TMH1 in fenoterol and trospium transport kinetics** Protein alignment of N-terminus and TMH1 of human and mouse OCT1 with amino acid differences highlighted and named (A). Effect of single substitutions of the amino acids that differ between human and mouse OCT1 on the uptake of 50 µM fenoterol (B, C) and 200 µM trospium (D, E). HEK293 cells transiently overexpressing human (green) and mouse (red) wild-type and mutant OCT1 were incubated with 50 µM fenoterol (B, C) or 200 µM trospium (D, E) for 2 min. OCT1-mediated uptake was calculated by subtracting the uptake of control cells (pcDNA5) from the uptake of OCT1-overexpressing cells. Shown are means and standard errors of the means of at least three independent experiments. *** P < 0.001 compared to wildtype in a Tukey’s post hoc analysis following one-way ANOVA.

Fig. S4. **Protein alignment of the TMH1 region in mammalian Oct1 orthologs** Human and mouse orthologs are highlighted in green and red, respectively. Codons 32 and 36 are highlighted in blue boxes.


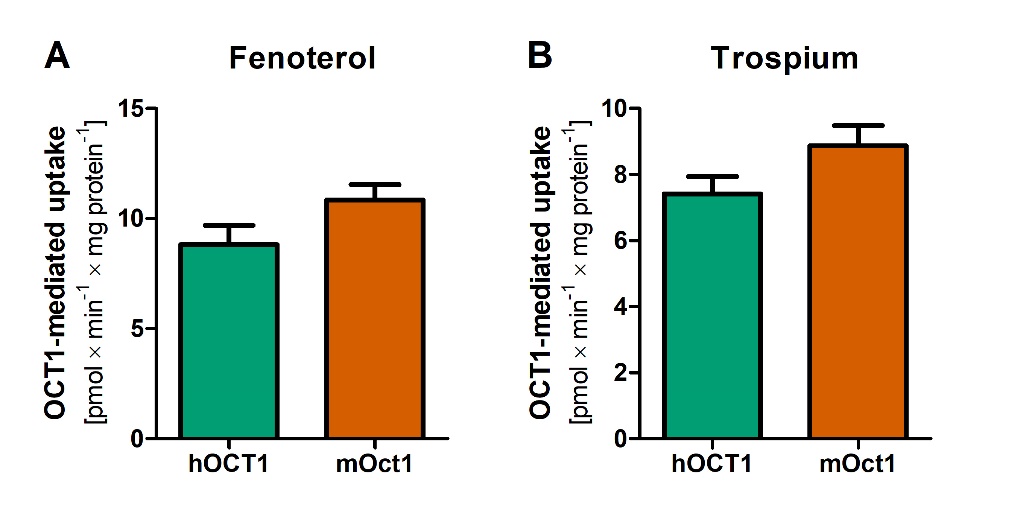


Fig. S5. **Comparison of fenoterol and trospium uptake at low clinically relevant concentrations between human and mouse OCT1** Plasma concentrations are estimated to be 2.18 nM for fenoterol (9) and 13.3 nM for trospium (39) based on clinical data. Shown is the uptake at the lowest concentration measured in Fig 2A and B, respectively. HEK293 cells stably overexpressing human (green) and mouse (red) OCT1 were incubated with 100 nM of (A) fenoterol and (B) trospium for 2 min. OCT1-mediated uptake was calculated by subtracting the uptake of control cells (pcDNA5) from the uptake of OCT1-overexpressing cells. Shown are means and standard errors of the means of at least six independent experiments.


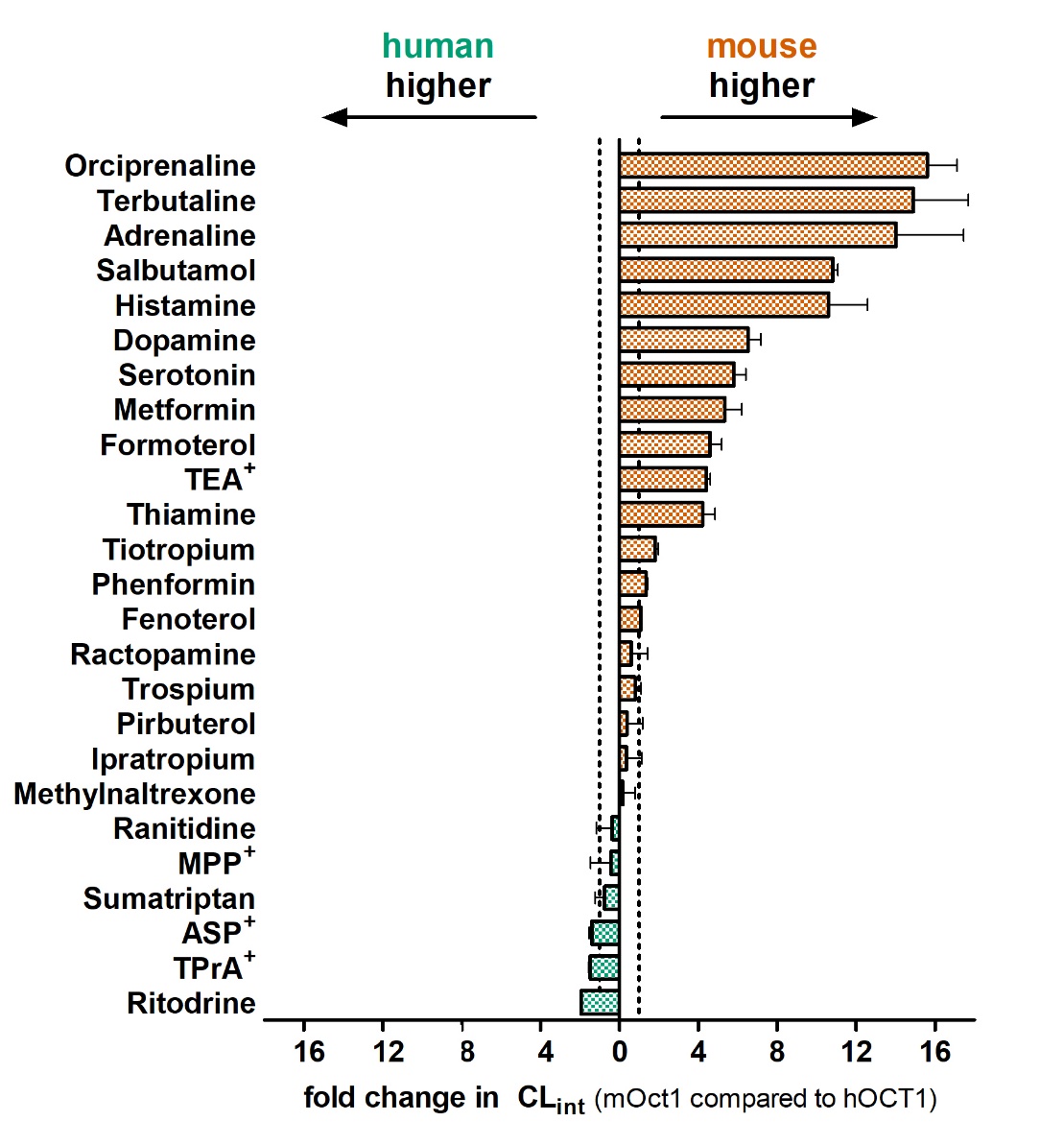


Fig. S6. **Comparison of calculated intrinsic clearance (CL_int_) between human and mouse OCT1 orthologs** Shown are the fold change in CL_int_ between human and mouse OCT1 for 25 drugs, model, and endogenous compounds. Isobutyryl-L-carnitine (IBC), choline, and noradrenaline could not be shown because the data for human OCT1 could not be fit to the Michaelis-Menten equation. Dashed line marks 2-fold difference. Shown are means and standard errors of the means of at least three independent experiments.


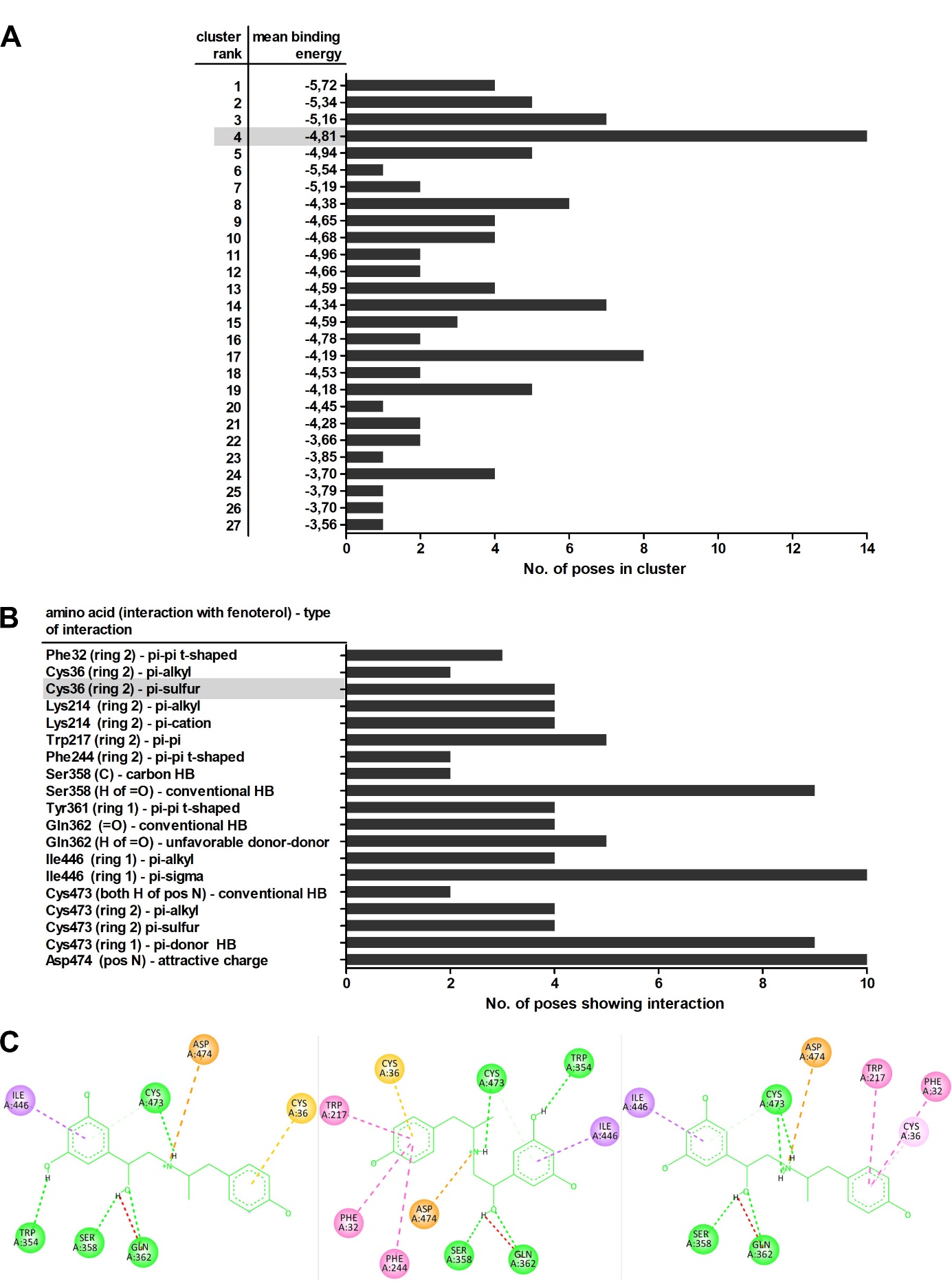


Fig. S7. **Summary of pose clustering, binding energy, and ligand-protein interactions after molecular docking of fenoterol into human OCT1** (A) Clustering of fenoterol docking poses and mean predicted binding energy of poses belonging to the cluster. Numbers of poses in each cluster are represented as bars. (B) Summary of ligand-protein interactions of docking poses in cluster 4 (highest populated cluster). Only interactions occurring more than once are shown. (C) Protein-ligand interactions of three selected poses from cluster 4.

Table S1. Parameters of LC-MS/MS detection

| **Analyte** | **Quantifier precursor ion to product ion (m/z)** | **Retention time [min]** | **Internal standard (IS)** | **IS precursor ion to product ion (m/z)** | **IS retention time [min]** | **Mobile phase [% organic solvent]*** | **Flow [µl/min]** | **Injection volume [µl]** |
| --- | --- | --- | --- | --- | --- | --- | --- | --- |
| Ranitidine | 315.1>130.2 | 4.2 | Ranitidine-d6 | 321.2>130.1 | 4.2 | 8 | 300 | 10 |
| Trospium | 392.1>164.0 | 2.9 | Trospium-d8 | 400.1>171.9 | 2.9 | 31.5 | 400 | 5 |
| Tiotropium | 391.8>152 | 2.51 | Trospium-d8 | 400.1>171.9 | 2.9 | 31.5 | 400 | 5 |
| Methylnaltrexone | 356.2>338.2 | 5.6 | Morphine | 286.2>201.1 | 3.2 | 8 | 300 | 10 |
| Fenoterol | 304.1>107.1 | 3.14 | Fenoterol-d6 | 310.3>109.1 | 3.11 | 12 | 450 | 10 |
| Formoterol | 345.2>149.1 | 4.0 | Fenoterol-d6 | 310.3>109.1 | 1.69 | 13.5 | 800 | 10 |
| Ritodrine | 289.0>271.0 | 3.83 | Fenoterol-d6 | 310.3>109.1 | 3.38 | 12 | 450 | 10 |
| Orciprenaline | 211.2>125.2 | 1.78 | Salbutamol | 240.5>222.0 | 2.65 | 2.7 | 800 | 10 |
| Ractopamine | 302.0>164.0 | 2.96 | Fenoterol-d6 | 310.3>109.1 | 1.7 | 13.5 | 800 | 10 |
| Terbutaline | 226.5>152.0 | 2.18 | Salbutamol | 240.5>222.0 | 2.67 | 3.6 | 800 | 10 |
| Pirbuterol | 240.0>166.9 | 1.6 | Fenoterol-d6 | 310.3>109.1 | 6.2 | 4.5 | 800 | 30 |
| Salbutamol | 240.5>222.0 | 2.67 | Terbutaline | 226.5>152.0 | 2.18 | 3.6 | 800 | 10 |
| Sumatriptan | 296.3>57.9 | 3.36 | Sumatriptan-d6 | 302.1>64.1 | 3.36 | 18 | 300 | 10 |
| Thiamine | 265.3>122.0 | 2.47 | Thiamine-d3 | 269.1>125.0 | 2.9 | 3 | 350 | 15 |
| Choline | 104.2>60.0 | 3.5 | Choline-d9 | 113.1>69.0 | 3.5 | 2.7 | 350 | 15 |
| Serotonine | 177.1>160.1 | 4.2 | Serotonine-d4 | 180.8>135.9 | 4.2 | 3 | 400 | 15 |
| Isobuturyl-L-carnitine | 232.0>85.0 | 4.36 | Isobuturyl-L-carnitine-d6 | 238.0>179.0 | 4.24 | 3 | 400 | 15 |
| Metformin | 130.1>71.0 | 1.66 | Buformin | 158.1>60.0 | 2.31 | 3 | 500 | 5 |
| Phenformin | 206.5>60.2 | 6.5 | Buformin | 158.1>60.0 | 2.57 | 3.6 | 450 | 5 |
| Buformin | 158.1>60.0 | 2.57 | Phenformin | 206.5>60.2 | 6.5 | 3.6 | 450 | 5 |
| Ipratropium | 332.3>166.2 | 5.42 | Atropine | 290.2>124.4 | 4.85 | 15 | 300 | 10 |
| Adrenaline | 184.2>166.2 | 2.24 | Buformin | 158.1>60.0 | 2.95 | 3 | 400 | 10 |
| Noradrenaline | 170.1>151.9 | 2.2 | Buformin | 158.1>60.0 | 2.95 | 3 | 400 | 10 |
| Histamine | 112.1>94.9 | 2.2 | Buformin | 158.1>60.0 | 3.4 | 3 | 350 | 5 |
| Dopamine | 154.1>137.2 | 2.97 | Buformin | 158.1>60.0 | 3.4 | 3 | 350 | 10 |
| TPrA^+^ | 187.1>145.0  187.1>115.0 |  | TEA | 131.0>100.8  131.0>86.9 | 2.0 | 30 | 400 | 15 |

m/z, mass-to-charge ratio

*six parts acetonitrile + one part methanol

Table S2. Primers used for site-directed mutagenesis

| **Primer** | **Sequence (5‘-> 3‘)** | **Amino acid substitution** |
| --- | --- | --- |
| I24L_hfor_new | TCCAGAAGCAAGCCTTCCTC**T**T**G**TTATGCCTGCTGTCGGCTGCC | Ile24Leu in hOCT1 |
| I24L_hrev_new | GGCAGCCGACAGCAGGCATAA**C**A**A**GAGGAAGGCTTGCTTCTGGA |  |
| L24I_mfor | CAGAAGCAAGCCTTCCTG**A**T**C**CTATGCCTGATCTCAGCT | Leu24Ile in mOct1 |
| L24I_mrev | AGCTGAGATCAGGCATAG**G**A**T**CAGGAAGGCTTGCTTCTG |  |
| L28I_hfor | TTCCTCATCTTATGCCTG**A**T**C**TCGGCTGCCTTTGCGCCC | Leu28Ile in hOCT1 |
| L28I_hrev | GGGCGCAAAGGCAGCCGA**G**A**T**CAGGCATAAGATGAGGAA |  |
| I28L_mfor | TTCCTGTTGCTATGCCTG**C**T**G**TCAGCTTCTTTAGCTCCC | Ile28Leu in mOct1 |
| I28L_mrev | GGGAGCTAAAGAAGCTGA**C**A**G**CAGGCATAGCAACAGGAA |  |
| A31S_hfor | TTATGCCTGCTGTCGGCT**T**C**T**TTTGCGCCCATCTGTGTG | Ala31Ser in hOCT1 |
| A31S_hrev | CACACAGATGGGCGCAAA**A**G**A**AGCCGACAGCAGGCATAA |  |
| S31A_mfor | CTATGCCTGATCTCAGCT**G**C**C**TTAGCTCCCATCTACGTG | Ser31Ala in mOct1 |
| S31A_mrev | CACGTAGATGGGAGCTAA**G**G**C**AGCTGAGATCAGGCATAG |  |
| F32L_hfor | TGCCTGCTGTCGGCTGCCTT**A**GCGCCCATCTGTGTGGGC | Phe32Leu in hOCT1 |
| F32L_hrev | GCCCACACAGATGGGCGC**T**AAGGCAGCCGACAGCAGGCA |  |
| L32F_mfor | TGCCTGATCTCAGCTTCTTT**T**GCTCCCATCTACGTGGGC | Leu32Phe in mOct1 |
| L32F_mrev | GCCCACGTAGATGGGAGC**A**AAAGAAGCTGAGATCAGGCA |  |
| C36Y_hfor | GCTGCCTTTGCGCCCATCT**AC**GTGGGCATCGTCTTCCTG | Cys36Tyr in hOCT1 |
| C36Y_hrev | CAGGAAGACGATGCCCAC**GT**AGATGGGCGCAAAGGCAGC |  |
| Y36C_mfor | GCTTCTTTAGCTCCCATCT**GT**GTGGGCATCGTTTTCCTG | Tyr36Cys in mOct1 |
| Y36C_mrev | CAGGAAAACGATGCCCAC**AC**AGATGGGAGCTAAAGAAGC |  |

Affected codons are underlined, changed bases are boldfaced
